# Targeting the ATX‐LPA Axis Overcomes TKI Resistance and Immunosuppression in Renal Cell Carcinoma via Dual Inhibition of AKT/mTOR and TBK1/IRF3 Pathways

**DOI:** 10.1002/advs.76352

**Published:** 2026-06-29

**Authors:** Jinchen Luo, Hansen Lin, Haoqian Feng, Lei Tan, Xi Liu, Yong Huang, Junjie Cen, Jiajie Chen, Xinwei Zhou, Mingjie Lin, Wuyuan Liao, Zheyu Ai, Minyu Chen, Yinghan Wang, Wei Chen, Junhang Luo, Yanping Liang

**Affiliations:** ^1^ Department of Urology The First Affiliated Hospital of Sun Yat‐Sen University Guangzhou China; ^2^ Urology Department The Fourth Affiliated Hospital of Guangzhou Medical University Guangzhou China; ^3^ Huadu District People's Hospital of Guangzhou Guangzhou China; ^4^ Huadu Institute of Medical Sciences Guangzhou China; ^5^ Guangxi Hospital Division of The First Affiliated Hospital Sun Yat‐sen University Nanning China; ^6^ Department of Pediatrics The First Affiliated Hospital of Sun Yat‐Sen University Guangzhou China; ^7^ Department of Urology Cancer Hospital Chinese Academy of Medical Sciences Shenzhen Center Shenzhen China; ^8^ Department of Laboratory Medicine The First Affiliated Hospital of Sun Yat‐Sen University Guangzhou China

**Keywords:** ENPP2/ATX, immunotherapy, Renal cell carcinoma (RCC), Tyrosine kinase inhibitor

## Abstract

**Background:**

Therapeutic resistance limits durable survival in advanced/metastatic renal cell carcinoma (RCC) treated with first‐line tyrosine kinase inhibitor (TKI) plus immune checkpoint inhibitor (ICI). We sought to define key resistance drivers and actionable targets.

**Methods:**

Integrated RNA sequencing of cabozantinib‐resistant RCC cells, lipid metabolomics, and PD‐L1 correlation analyses identified ENPP2 as a candidate driver. Its role in TKI resistance and survival signaling was validated by apoptosis, CCK‐8, and colony formation assays in vitro and by nude‐mouse xenograft models in vivo. ELISA, flow cytometry and tumor cell–T‐cell co‐culture assays were used to dissect ENPP2‐dependent CD8+ T‐cell dysfunction. The therapeutic benefit of pharmacologic ATX inhibition combined with standard TKI–ICI regimens was tested in RCC patient‐derived xenograft models.

**Results:**

The ATX–LPA axis conferred TKI resistance via constitutive AKT/mTOR activation and promoted immune evasion by upregulating PD‐L1 through TBK1/IRF3 signaling, thereby impairing intratumoral CD8+ T‐cell function. ENPP2 enhanced PD‐L1 transcription by facilitating IRF3 nuclear translocation and its direct recruitment to the CD274 promoter. ATX inhibition improved the antitumor efficacy of TKI–ICI therapy in preclinical models.

**Conclusions:**

Targeting the ATX–LPA axis represents a promising strategy to overcome resistance to current TKI–ICI combinations.

## Abstract 2

ENPP2 (Autotaxin) catalyzes the conversion of LPC to LPA. In the tumor microenvironment, LPA engages LPAR1 on renal cancer cells to activate TBK1. This signaling bifurcates to: (1) stimulate the AKT–mTOR cascade, conferring resistance to tyrosine kinase inhibitors (TKIs); and (2) induce IRF3 phosphorylation and nuclear translocation. Nuclear IRF3 transactivates the CD274 promoter, upregulating surface PD‐L1. Subsequent PD‐L1/PD‐1 engagement, further exacerbated by the accumulation of immunosuppressive LPA, triggers T‐cell exhaustion (characterized by increased TIM‐3/LAG‐3 and impaired GZMB/TNF‐α secretion). Conversely, pharmacological blockade of ENPP2 with GLPG1690 abrogates LPA synthesis, silencing downstream LPAR1–TBK1 signaling and alleviating extracellular suppressive stress on T cells. This intervention downregulates PD‐L1, restores TKI sensitivity, and reinvigorates cytotoxic T‐cell effector functions.

## 1 Introduction

Renal cell carcinoma (RCC) represents a prevalent malignancy of the urinary system, accounting for over 80,000 new cases and more than 14,000 deaths annually in the United States alone [1]. This malignancy is characterized by von Hippel‐Lindau (VHL) gene mutations that drive pathological angiogenesis, extensive leukocyte infiltration, and intrinsic resistance to conventional therapies, including chemotherapy and radiotherapy [2]. Over the past few decades, the therapeutic landscape for RCC has evolved significantly, with angiogenesis‐targeting tyrosine kinase inhibitors (TKIs) and immune checkpoint inhibitors (ICIs), primarily targeting PD‐1, significantly improving patient survival outcomes. Currently, combination therapies involving TKIs and ICIs have emerged as first‐line clinical regimens for RCC management [3, 4, 5]. However, a substantial proportion of patients exhibit either primary resistance to these therapies or develop acquired resistance during treatment, resulting in suboptimal long‐term disease control [6]. These challenges highlight the urgent need to identify molecular drivers of therapeutic resistance and immune evasion in RCC, which will facilitate the discovery of novel therapeutic targets for achieving durable clinical responses.

RCC is a highly immunogenic malignancy. Large‐scale mass cytometry analyses of clear cell RCC (ccRCC) tumors have demonstrated that T cells constitute the predominant immune cell population within the tumor microenvironment (TME), accounting for an average frequency of 51% [7]. Paradoxically, despite this robust T‐cell infiltration, tumor‐infiltrating lymphocytes (TILs) fail to mount effective antitumor responses and are even associated with poor clinical outcomes [8, 9]. This striking observation strongly suggests an immunosuppressive state of T cells within the RCC TME. Previous studies have revealed that antiangiogenic TKIs or mTOR inhibitors may upregulate tumor PD‐L1 expression, providing mechanistic insights into treatment resistance and supporting the rationale for combination therapies [10, 11]. Lipid metabolic reprogramming plays a pivotal role in tumor progression and therapeutic resistance [12, 13]. A hallmark of ccRCC is the accumulation of lipid droplets (LDs), driven in part by VHL gene mutations [12]. Emerging evidence indicates that lipid metabolic rewiring contributes to acquired drug resistance in tumors [14]. Recent studies have specifically linked dysregulated lipid metabolism to increased resistance to sunitinib and pazopanib in RCC patients [15, 16], highlighting the critical role of lipid metabolism in the development of acquired resistance mechanisms. Several studies have also highlighted the role of metabolic reprogramming in the resistance to immune responses of RCC, suggesting that combining PD‐1 inhibitors with targeted modulation of metabolic pathways could enhance immunotherapy efficacy. However, the specific impact of lipid metabolism in the RCC tumor microenvironment, particularly in relation to anti‐tumor immunity, remains poorly understood [17].

Ectonucleotide pyrophosphatase/phosphodiesterase 2 (ENPP2), encoding the secreted enzyme autotaxin (ATX), catalyzes the hydrolysis of lysophosphatidylcholine (LPC) to lysophosphatidic acid (LPA), which activates downstream signaling through at least six G protein‐coupled receptors (LPAR1‐6) [18]. ATX has been recognized as a “gatekeeper” of LPA signaling, with the majority of its biological functions attributed to its role in LPA biosynthesis [19]. The ATX‐LPA axis has been implicated in tumor proliferation, invasion, angiogenesis, and acquired resistance to multiple therapeutics including sunitinib and cisplatin [20, 21, 22, 23, 24, 25]. Recent studies have further emphasized the dual role of ATX‐LPA signaling axis in modulating the tumor immune microenvironment. While multiple reports demonstrate that ATX‐LPA signaling facilitates immune evasion by impairing T‐cell functionality or disrupting T cell infiltration into the tumor site [26, 27, 28], synergistic antitumor effects have also been observed when combining LPA with conventional chemotherapeutics in colorectal cancer (CRC) [29]. These conflicting findings highlight the complex and context‐dependent role of ATX‐LPA signaling in tumor immune microenvironment.

Notably, the specific impact of the ATX‐LPA axis on the immune landscape of RCC, and its mechanistic underpinnings, remain poorly characterized. Our study addresses this gap by investigating how the ATX‐LPA axis contributes to both therapeutic resistance and immune evasion in RCC. We found that ENPP2, the key enzyme in LPA biosynthesis, activates the AKT/mTOR pathway, driving resistance to TKI treatment, and simultaneously induces PD‐L1 expression via the TBK1/IRF3 signaling pathway. Importantly, we demonstrate that ENPP2 promotes PD‐L1 transcription through IRF3 nuclear translocation and direct recruitment to the CD274 promoter. Additionally, we provide evidence that targeting ENPP2 with pharmacological inhibitors reverses both TKI resistance and immune suppression, restoring CD8+ T cell function. These findings open new avenues for combining ENPP2 inhibition with existing TKI‐ICI therapies to overcome resistance and enhance therapeutic efficacy in advanced RCC.

## 2 Materials and Methods

### 2.1 Patients and Specimens

Human renal cell carcinoma (RCC) specimens were prospectively collected from treatment‐naïve patients undergoing nephrectomy at the Department of Urology, the First Affiliated Hospital of Sun Yat‐sen University (Guangzhou, China). This study protocol received ethical approval from the Medical Ethics Committee of the First Affiliated Hospital, Sun Yat‐sen University, with written informed consent obtained from all participants in strict accordance with the Declaration of Helsinki principles. RCC tissues treated with RNAlater and stored at −20°C were used for qRT‐PCR analyses. RCC tissues stored at −80°C were used for Western blotting analyses. The baseline information for the public cohorts used in our study was shown in Table S1.

### 2.2 Cell Culture and Cell Lines

The human RCC cell lines [786‐O (RRID: CVCL_1051), ACHN (RRID: CVCL_1067) were purchased from ATCC. The ACHN were cultured in DMEM. The 786‐O were cultured in RPMI1640 medium. All media were obtained from Gibco and supplemented with 1% penicillin–streptomycin (Biosharp Biotechnology) and 10% FBS (BioChannel). All cell lines were examined with short tandem repeat profiling by the vendors and routinely tested for Mycoplasma infection.

Cabozantinib‐resistant RCC models were established using 4‐week‐old male BALB/c‐Nu mice maintained under SPF conditions, adapting an in vivo serial passaging protocol recently described by our group (Advanced Science, 2025; https://doi.org/10.1002/advs.202506367) [30]. Subcutaneous inoculation with 786‐O or ACHN cells (1 × 10^7^ cells/100 µL PBS) was performed, with cabozantinib treatment initiated two weeks post‐inoculation when tumor volumes reached 200 mm^3^. Mice received daily oral gavage of 40 mg/kg cabozantinib (Selleck, S4001) formulated in 10% DMSO / 90% corn oil vehicle for six consecutive weeks. Following chronic drug exposure, tumors were aseptically resected, fragmented into 1 mm^3^ explants, and surgically transplanted into secondary recipient mice under continued cabozantinib or vehicle treatment. This serial passaging protocol was repeated through three generations to enrich therapy‐resistant populations. Terminal third‐passage tumors underwent enzymatic dissociation using ACCUMAX solution (Innovative Cell Technologies) with 30‐min incubation at 37°C with continuous shaking. After centrifugation at 300×g, cell suspensions underwent three cycles of differential adherence to eliminate fibroblasts, with purified epithelial cells plated at 5 × 10^5^ cells/well in 6‐well culture plates. The resultant cabozantinib‐resistant sublines were designated 786‐R and ACHN‐R. Functionally, TKI resistance in these sublines was defined in vitro by their retained capacity for cell survival under escalating drug concentrations. Viability assays confirmed that the isolated 786‐R and ACHN‐R cells exhibited significantly higher half‐maximal inhibitory concentrations (IC50) compared to their treatment‐naïve parental counterparts.

For ENPP2 knockout cell lines, 786 and ACHN cells expressing CRISPR‐Cas9 were seeded in 6‐cm dishes. Upon reaching 20%–30% confluence, cells were transfected with sgRNA (sequence: UCCAACGGCAAAAGUGAACA). At 72 h post‐transfection, the top 5% fluorescent cells were isolated via fluorescence‐activated cell sorting (FACS) and plated into 96‐well plates at a density of one cell per well. Single‐cell clones were expanded stepwise into 24‐well and 6‐well plates. ENPP2 knockout efficiency was validated by Western blotting.

### 2.3 Plasmid, RNA Interference, and Lentivirus Construction

The siRNA, shRNA lentivirus, and overexpression lentivirus were constructed, identified, and provided by Ribio (China).

The detailed sequence information is provided in Table S2.

### 2.4 Antibodies and Reagents

The catalog numbers of all Western blot and flow cytometry antibodies used in this study are provided in Table S3.

### 2.5 Real‐Time RT‐PCR

Total RNA was extracted from tumor tissues or cell lines using TRIzol (Invitrogen). Reverse transcription of total RNA was performed using the Reverse Transcription Master Mix (EZBioscience). Quantitative RT‐PCR (qRT‐PCR) was conducted with SYBR Green Real‐Time PCR Master Mix (EZBioscience) using a Roche LightCycler480 instrument. The amplification parameters were set at 95°C for 30 s, 58°C for 30 s, and 72°C for 30 s (for a total of 35 cycles). The expression levels of all target genes were normalized to the control gene GAPDH. The following primers were used: *GAPDH*: forward, 5′ ‐GGAGCGAGATCCCTCCAAAAT – 3′, reverse, 5′—GGCTGTTGTCATACTTCTCATGG – 3′; *ENPP2*: forward, 5′—ACTTTTGCCGTTGGAGTCAAT – 3′, reverse, 5′—GGAGTCTGATAGCACTGTAGGA – 3′. *CD274*: forward, 5′—TGGCATTTGCTGAACGCATTT – 3′, reverse, 5′—TGCAGCCAGGTCTAATTGTTTT – 3′.

### 2.6 Western Blotting

The cells were lysed with RIPA lysis buffer (Beyotime, China) supplemented with a protease inhibitor cocktail (CoWin Biosciences, China) on ice for 15 min. The protein concentration of each sample was measured using the Pierce BCA Protein Assay Kit (ThermoFisher, USA) and detected at a wavelength of 562 nm. After denaturation with 1x sodium dodecyl sulphate (SDS) loading buffer, equivalent proteins were electrophoresed using SDS‐polyacrylamide gel electrophoresis (SDS241 PAGE) and transferred to a 0.45 µm polyvinylidene difluoride (PVDF) membrane (Merck Millipore, Billerica, MA, USA). After blocking, the membranes were incubated with primary antibodies at 4°C for more than 12 h. Primary antibodies for western blotting were showed in Table S3. The membranes were incubated with secondary antibodies (HRP‐conjugated anti‐rabbit/mouse IgG, Proteintech) for 1 h at room temperature. Protein bands were detected by chemiluminescence (Tanon).

### 2.7 Cell Fractionation

ccRCC cells were harvested, washed, and resuspended in cold PBS. Cytoplasmic and nuclear protein fractions were separated using a Nuclear and Cytoplasmic Protein Extraction Kit (#P0028, Beyotime) according to the manufacturer's protocol, and fractions were subsequently analyzed via western blotting.

### 2.8 CCK‐8 and Colony Formation Assays

The proliferation ability of the indicated cells was measured using the Cell Counting Kit‐8 (CCK‐8; Dojindo, Tokyo, Japan) according to the manufacturer's protocol. Briefly, a total of 1000 cells were seeded per well in a 96‐well plate, 10 µL of CCK‐8 solution was added to each well and incubated for 2 h at 37°C. The optical density (OD) was measured at 450 nm using a spectrophotometer. Each experiment was performed in triplicates. For IC50 calculation, 5000 cells per well were treated with the indicated concentrations of cabozantinib for 48 h and IC50 was calculated using GraphPad Prism 10 (GraphPad Software).

For colony formation assays, 1000 cells were seeded in a 6‐well plate in 2 mL of fresh medium and cultured for 1–2 weeks. The colonies were fixed with 4% paraformaldehyde (PFA) for 20 min at room temperature and then stained with 0.4% crystal violet. The number of colonies was counted and analyzed using ImageJ software.

### 2.9 Transwell Assays

5 × 10^4^ 786‐O or ACHN cells were resuspended in 200 µL of serum‐free medium in a 24‐well transwell chamber (Corning, NY, USA). For invasion assay, the chamber membrane was precoated with Matrigel (Corning, NY, USA) at 37°C for 1 h. The lower chamber was supplemented with medium containing 10% FBS as a chemoattractant. After incubation for 8 h for the migration assay and 16 h for the invasion assay, cells that migrated or invaded the lower surface were fixed with 4% PFA (Beyotime, China) for 30 min and stained with 0.4% crystal violet (Beyotime, China) for 30 min and counted under a microscope.

### 2.10 EdU Experiment

The experiment involved seeding cells into 6‐well plates (with coverslips) in amounts of 3 × 10^5^ cells/well and incubating them for 48 h after treatment with different substances. The cultured cells were then permeabilized with EdU (5‐ethynyl‐2'‐deoxyuridine) using the BeyoClick EdU‐594 Cell Proliferation Assay Kit (Beyotime, China). The final concentration of EdU was adjusted to 10 µm (working solution). RCC cells were treated with the working solution and incubated for 1–2 h. After that, the cells were fixed with 4% paraformaldehyde (Beyotime, China) by immersion for 15 min, and rinsed twice with 1 × PBS solution for 5 min each time at room temperature. Then, 0.3% Triton X‐100 PBS was then used to permeabilize the cells. The cells were rinsed twice with 1 × PBS solution for 5 min each time at room temperature, followed by incubation with the reaction solution for 30 min. The reaction solution was added to the cells and incubation was 30 min. DAPI dye was used to stain the cell nuclei, and the wavelength of 594 nm was used to detect EdU. The images were photographed using an automated inverted fluorescence microscope (Olympus, Japan) or Confocal Microscope (Olympus, Japan).

### 2.11 Immunofluorescence Staining

Cells were seeded in confocal dishes and cultured for 24 h. Following phosphate‐buffered saline (PBS) washes, cells were fixed with 4% PFA for 15 min at room temperature and permeabilized with 0.3% Triton X‐100 for an additional 15 min. Blocking was performed using 5% Bovine Serum Albumin (Sigma‐Aldrich) for 1 h. Primary antibodies targeting AKT (#4685, CST), TBK1 (#38066, CST) or IRF3 (#11904, CST,) were applied and incubated overnight at 4°C. After washing, Alexa‐Fluor‐conjugated secondary antibodies (Invitrogen) were added for 1 h at room temperature. Nuclear staining was performed using 4,6‐diamidino‐2‐phenylindole (D3571, Invitrogen). Imaging was conducted with an OLYMPUS FV3000 confocal microscope (Olympus). Colocalization analysis was performed using ImageJ with the Colocalization Finder plugin. The degree of colocalization was quantitatively evaluated by calculating the Pearson's correlation coefficient (PCC) and Manders’ overlap coefficients (M1 and M2), as provided by the plugin.

### 2.12 Luciferase Reporter Analysis

For the PD‐L1 promoter analysis, different lengths of the PD‐L1 promoter and corresponding mutant fragments were cloned into the PGL3 basic vector. The ratio of the luminescence of the firefly luciferase to that of the Renilla luciferase was calculated using the Dual Luciferase Reporter Gene Assay Kit (RG027, Beyotime).

### 2.13 Enzyme‐Linked Immunosorbent Assay (ELISA)

The ELISA kit for LPA tests was purchased from cloud‐clone corp (#CEK623Ge, USCN, China). The detailed procedures were performed according to the manufacturer's protocol. Briefly, 50 µL of cell culture medium and 50 µl of detection reagent A were added into each well, incubated for 1 h at 37°C and then washed with wash buffer three times. Then, 100 µL of detection reagent B was added to each well, followed by incubation at 37°C for 30 min and washing with wash buffer three times. Finally, 90 µL of substrate solution was added to each well, and 50 µL of stop solution was supplemented 15 min later. The absorbance at 450 nm of each well was measured immediately.

Murine blood samples were obtained via serum separator tubes prior to euthanasia. The samples were allowed to clot at room temperature for 1 h, followed by centrifugation at 3,000 × g for 10 min. Serum was aliquoted into fresh cryovials and stored at −80°C until analysis. Serum concentrations of IFN‐γ and TNF‐α were quantified using human‐specific ELISA kits (Elabscience, USA) according to the manufacturer's protocols.

### 2.14 Immunohistochemistry (IHC)

Paraffin sections of ccRCC and normal tissues were first deparaffinized and hydrated. Antigen retrieval was then performed using microwave heating with citrate buffer (pH 9.0). To block endogenous peroxidase activity, the sections were incubated with 0.3% H_2_O_2_ for 10 min. Afterward, the sections were treated with the primary antibody at 4°C for 12 h, followed by incubation with the secondary antibody at room temperature for 1 h. Visualization was achieved using peroxidase and 3,3′‐diaminobenzidine tetrahydrochloride (DAB), after which the sections were counterstained with hematoxylin and mounted using non‐aqueous mounting medium. Images were captured using the KF‐PRO‐020 Digital Slice Scanner.

### 2.15 T‐Cell Separation, Activation, and Culture

Peripheral blood mononuclear cells (PBMC) were isolated from healthy donors using FicollPaque (17‐5442‐02, GE Healthcare) density gradient centrifugation following the manufacturer's instructions, and PBMCs were washed twice with PBS. Primary human CD8+ T cells were purified using a CD8+ T‐cell Isolation Kit (Miltenyi Biotec, 130‐096‐495) following the manufacturer's instructions. For human T‐cell activation, ImmunoCult Human CD3/CD28/CD2 T‐cell activator (Cat: 10970; STEMCELL Technologies, USA) was used to activate T cells. T‐cell medium supplemented with Recombinant Human IL‐2 Protein (Cat: 202–1 L‐050, R&D, USA) was used for human T‐cell culture.

### 2.16 Co‐Culture Assay and LDH Assay

Gradient co‐culture ratios (1:5, 1:10, 1:15, 1:20) of CRISPR‐engineered RCC cells (786‐sgNC, 786‐sgENPP2, ACHN‐sgNC, ACHN‐sgENPP2) and activated human T cells from healthy donors were established in 12‐well plates. The supernatants were collected for the LDH assay (Cat: 88954, Thermo Fisher, USA) according to the manufacturer's protocol. T cells were collected and stained with antibodies for further flow cytometry analysis.

### 2.17 Flow Cytometry

For apoptosis analysis, cells were collected and evaluated by the Annexin V‐APC/propidium iodide (PI) apoptosis kit (AP‐107, Multi Science) according to the manufacturer's instructions. Samples were analyzed with a Beckman CytoFLEX Flow cytometer (Beckman Coulter, RRID:SCR_019627), and FlowJo 10 software was used to analyze the data.

Tumor samples were collected and digested into single‐cell suspensions. Subsequently, tumor infiltrating lymphocytes (TILs) were extracted. After washing twice with DPBS, the cells were used for subsequent experiments. For cell surface marker analysis, samples were first stained with Zombie UV (423108, Biolegend) at room temperature for 15 min before staining with different antibodies. The cells were then resuspended and stained with specified antibodies at 4°C for 30 min. Intracellular fixation and permeabilization were performed using the buffer set from Invitrogen. For the detection of intracellular effector molecules and cytokines, T cells were treated with RPMI1640 medium containing Brefeldin A for 4–6 h. The Stained cells were detected using a BD LSRFortessa, and data analysis was conducted with FlowJo software (version 10.8.1). The antibodies that used were shown in Table S3.

### 2.18 Luciferase Reporter Analysis

In brief, different full‐length and truncated genes were inserted into the PGL3 vector. 293 T cells (3 × 10^3^ cells/well) were seeded into 96‐well plates. The ratio of the luminescence of the firefly luciferase to that of the *Renilla* luciferase was 5:1. The luciferase activity of each well was determined with a Dual Luciferase Reporter Assay Kit (Beyotime) on a Varioskan LUX machine (ThermoFisher, MA, USA). The luciferase activity was normalized to the Renilla control.

### 2.19 Isolation of PDX RCC Cells

Tumor tissues were isolated and washed with cold PBS (supplemented with 2% penicillin–streptomycin solution). The tumors were then cut into pieces and digested in DMEM containing 0.002% DNase I (Stemcell, Canada), 0.01% hyaluronidase (Stemcell, Canada), 0.2% collagenase IV (Stemcell, Canada) at 37°C for 60 min with continuous shaking at 200 rpm. The digestion was centrifuged at 300 g, and the supernatant was filtered through a 40 µm cell strainer (352340, Corning). The cell suspension was cultured in a six‐well plate with DMEM containing 10% FBS and 1% penicillin–streptomycin solution.

### 2.20 Generation of DCs and Tumor‐Specific CD8+ T Cells

For the generation of DCs, mononuclear cells were obtained from the peripheral blood of HLA‐A2+ healthy donors and cultured in VIVO medium (04‐418Q, Lonza) supplemented with 100 ng mL‐1 GM‐CSF and 30 ng mL‐1 IL‐4 (PeproTech, USA). The medium and cytokines were replaced every 3 d. At Day 6, DCs were mature, and they were stimulated with 10 ng mL‐1 TNF‐α (PeproTech) for 24 h. The DCs were then pulsed for another 24 h with tumor lysates from HLA‐A2+ patients by freeze‐thawing with liquid nitrogen. To generate tumor‐specific CD8+ T cells, CD8+ T cells were isolated from the peripheral blood of the same donors as described above. The isolated CD8+ T cells were cocultured with mature DCs at a ratio of 5:1 in VIVO medium (Lonza, Switzerland) containing 25 IU mL‐1 IL‐2 (PeproTech) for 6 d to induce tumor‐specific T cells.

### 2.21 In Vivo Experiments

Animal care and in vivo mouse experiments were conducted with the approval of the Institutional Animal Care and Use Committee of SYSU, according to established guidelines. All animal research programs were approved by the Animal Ethics Committee of SYSU. Male BALB/c nude mice, immunocompetent wild‐type BALB/c mice, and immunocompromised NCG mice were purchased from GemPharmatech (Nanjing, Jiangsu, China). All animals were housed under specific pathogen‐free (SPF) conditions and randomly allocated to experimental groups prior to modeling or treatment. For the xenograft mouse model, 1 × 10^7^ 786‐O cells were injected subcutaneously into the flanks of 4‐week‐old BALB/c nude mice. The tumor size was measured weekly using the following formula: Tumor volume = 0.5 × length × width^2^. Six weeks later, mice were sacrificed, and tumors were excised for volume and weight determination. To validate the immune dependency of ENPP2‐mediated tumor progression, a syngeneic subcutaneous model was established. Immunocompetent BALB/c mice were subcutaneously inoculated with 5 × 10^5^ ENPP2‐depleted (shENPP2) Renca cells suspended in 100 µL of PBS. For in vivo T‐cell depletion, mice received intraperitoneal injections of specific neutralizing antibodies (200 µg per mouse in 100 µL PBS): CD8‐depleting antibody (BioXCell, Cat# BP0117), CD4‐depleting antibody (BioXCell, Cat# BP0003‐1), a combination of both, or an IgG isotype control. The initial antibody administration was performed one day prior to tumor engraftment, followed by consecutive maintenance injections every 3 days to ensure sustained depletion. For the orthotopic syngeneic RCC model, luciferase‐expressing Renca cells (with or without ENPP2 depletion) were orthotopically implanted into the subcapsular of the kidney of BALB/c mice. Tumor progression was monitored using in vivo bioluminescence imaging.

For the patient‐derived xenograft (PDX) mouse models, ccRCC specimens were obtained from patients diagnosed with ccRCC who underwent surgery in FAH SYSU. Our study was conducted with the informed consent of the patients and was approved by the Institutional Review Board. Fresh tumor tissues were cut into fragments of 1–2 mm^3^ and subcutaneously transplanted into NCG mice. When the tumor volume reached 200 mm^3^, the tumors were separated and cut into 1–2 mm^3^ pieces. The tumor pieces were transplanted into the next‐generation NCG mice. After three passages, a stable RCC PDX model was established. When the tumor volume reached 100 mm^3^, the mice were randomly allocated into following eight groups: control group, cabozantinib treatment group, immunotherapy (IT) group, ENPP2 inhibitor (EI) treatment group, EI + cabozantinib treatment group, EI + IT treatment group, cabozantinib + IT (combination) treatment group, EI + cabozantinib + IT treatment group.

Human DCs and tumor‐specific CD8+ T cells were generated as described above. For adoptive T cell transfer, tumor‐specific CD8+ T cells (2.5 × 10^6^ cells per mouse) and DC cells (0.5 × 10^6^ cells per mouse) were injected via tail vein to rebuild the human immune system. Mice were then administered ENPP2‐inhibitor (60 mg/kg once a day). For immunotherapy treatment, mice were rebuilded the human immune system and were intraperitoneally injected with anti‐PD‐1 antibody (BioXcell, USA) or IgG isotype control (BioXcell, USA) (5 mg/kg every 4 day for a total of seven times). The palpable tumor weight was measured every week. The tumor volume (mm^3^) was calculated as follows: tumor volume = 0.5 × length × width^2^. The mice were sacrificed and the tumors were separated surgically for IHC staining.

### 2.22 Bioinformatic Analysis

The lipid metabolism‐related gene sets were obtained from MsigDB, Reactome, GO and KEGG database, total 2279 genes, the full list of genesets is provided in Table S4. For bulk RNA data, the clinical and pathological information, as well as bulk RNA sequencing data of 521 ccRCC patients, were obtained from the Cancer Genome Atlas (TCGA) database (https://xenabrowser.net/datapages/). Transcripts per kilobase million (TPM) values were derived from RNA sequencing counts. The patient information for immunotherapy was obtained from the Javelin 101 cohort. To objectively define high‐ and low‐expression groups for survival and downstream differential analyses, the optimal biological thresholds for continuous gene expression variables were determined using the surv_cutpoint function within the survminer R package. ssGSEA quantifies the proportions of infiltrating immune cells based on the expression of marker genes. Gene Ontology (GO) and Kyoto Encyclopedia of Genes and Genomes (KEGG) analyses were conducted to explore the biological functions and pathways associated with the differentially expressed genes. The HALLMARK gene set was from the MSigDB database and the “clusterProfiler” package was used for Gene Set Enrichment Analysis (GSEA) enrichment analysis on differentially expressed genes between high‐and low‐expression groups. “GseaVis” R package was used for visualization.

Single‐cell RNA sequencing (scRNA‐seq) datasets were obtained from the following sources: GSE207493 [31], and Obradovic et al. (Table S5) [32]. Prior to dataset integration, we performed initial quality control (QC) to remove low‐quality cells. Cells were excluded if they met any of the following criteria: fewer than 300 or more than 5000 detected genes, less than 500 UMIs, mitochondrial gene content exceeding 20%, or hemoglobin gene content greater than 1%. We then applied DoubletFinder to identify and filter potential doublets. After QC, datasets were merged, and only genes expressed in both datasets were retained to minimize batch effects arising from differences in sequencing depth. Data normalization was performed using the NormalizeData function. A set of 2,000 highly variable genes (HVGs) was identified using the FindVariableFeatures function, followed by feature scaling to unit variance across all genes. For clustering and dimensionality reduction, principal component analysis (PCA) was conducted on the top 50 principal components (PCs), with batch effect correction implemented using the Harmony package. Cell clustering was performed using the Louvain algorithm, and Uniform Manifold Approximation and Projection (UMAP) was applied for visualization. We annotated six major cell types based on well‐established marker genes: T lymphocytes and NK cells: CD3D, CD3E, CD8A, CD4, NKG7, KLRD1. B lymphocytes and plasma cells: CD79A, CD79B, CD19, MS4A1, BCL11A. Myeloid cells: C1QA, C1QB, C1QC, CD68, ITGAX, CD14. Endothelial cells: PECAM1, RAMP2, AQP1, CLDN5. Fibroblasts: COL1A1, COL3A1, ACTA2, PDGFRB. Epithelial cells: EPCAM, CA9, NDUFA4L2, NNMT, KRT18, ANGPTL4. Among the epithelial cells, a subset exhibiting significantly higher expression of CA9 and NDUFA4L2 was identified as malignant renal clear cell carcinoma (RCC) cells.

For CD8+T cell annotations, first CD8+T cells were extracted from the whole T & NK cluster. Putative contaminating cells were removed on the basis of the markers described above. We annotated seven subtypes of CD8+T cells based on several previous studies, including CCR7, TCF7, IL7R for central memory T cells; ZNF683, KLRD1, ITGAE for resident memory T cells; HSPA1B, HSPA6, DNAJB4, BAG3 for stress‐response T cells; CXCL13, PDLIM4 for CXCL13+ T cells; PDCD1, CTLA4, LAG3 for exhausted T cells; TCF7, PDCD1, LAG3 for progenitor exhausted T cells; CXCR4, ANXA1, NFKBIA, GZMA for effector T cells [33, 34, 35, 36].

To recover dropout events in the gene expression data, we applied MAGIC (V2.0.3; Rmagic), an imputation algorithm based on Markov diffusion processes [37]. Prior to imputation, only genes detected in at least 10 cells were retained to ensure robustness. The raw count matrix was then log‐normalized before imputation. Imputation was performed using the following parameters: knn = 15, t = 3, and decay = 1. The resulting imputed expression matrix was integrated into the Seurat object for downstream analyses. Importantly, imputed values were used solely for gene expression visualization and correlation analysis, whereas all differential expression tests and clustering steps were conducted on the original, non‐imputed counts. This strategy minimizes the risk of introducing false positives due to over‐smoothing during imputation.

Pathway activity scores in malignant cells were assessed using the R package “AUCell” (V2.4.0). We curated six biologically relevant gene sets associated with cancer progression: Hypoxia, Epithelial‐Mesenchymal Transition (EMT), Angiogenesis, Inflammation, Apoptosis, and Stemness. These gene sets were derived from CancerSea dataset.

### 2.23 Statistical Analyses

Statistical analyses were performed using R version 4.3.2 or GraphPad Prism 10 software. Data are presented as the mean ± standard deviation (SD). All in vitro experiments were performed with at least three independent biological replicates. Data were analyzed for normality before comparisons. For comparisons between two groups, statistical significance was determined by two‐tailed Student's *t*‐test. For multiple comparisons, one‐way ANOVA with Tukey's post hoc test was used. For the survival analyses, OS was defined as the time from the operation to the date of death for any reason. Kaplan‐Meier survival curves were plotted with log‐rank tests. Correlation analyses were performed using Pearson's correlation (continuous variables) or Spearman's correlation (discontinuous variables). A *p* value less than 0.05 was considered significant. Statistical significance was shown as *(*p* < 0.05), ** (*p* < 0.01) or *** (*p* < 0.001).

## 3 Results

### 3.1 Identification of ENPP2 as a Key Lipid Metabolism Gene Associated With Malignant Phenotypes in RCC

To elucidate lipid metabolism–related mechanisms underlying tyrosine kinase inhibitor (TKI) resistance in renal cell carcinoma (RCC), we systematically integrated 2,279 lipid metabolism–associated genes curated from the KEGG, GO, Reactome, and MSigDB databases. Concurrently, TKI‐resistant RCC cell lines were established in vivo, and RNA sequencing (RNA‐seq) was performed to identify TKI resistance‐related genes. Integrating these data with survival information from the TCGA‐KIRC cohort enabled the identification of genes linked to both TKI response and patient prognosis. The intersection of these datasets yielded six candidate genes: EDN1, EGR1, ENPP2, ACOT11, PLA2G4C, and SDR42E1 (Figure 1A,B). Further correlation analyses in the TCGA and E‐MTAB‐1980 cohorts revealed that only PLA2G4C and ENPP2 exhibited consistent positive associations with PD‐L1 expression across both cohorts (Figure S1A,B) (though ENPP2 did not reach statistical significance in the E‐MTAB cohort). Single‐sample gene set enrichment analysis (ssGSEA) demonstrated that high ENPP2 expression correlated with significantly reduced activated CD8+ and CD4+ T‐cell infiltration, whereas PLA2G4C expression showed no association with CD8+ T cells abundance (Figure S1C,D). Given the critical role of CD8+ T cells in antitumor immunity, ENPP2 was prioritized for mechanistic exploration of its immunomodulatory functions in RCC.

**FIGURE 1:**
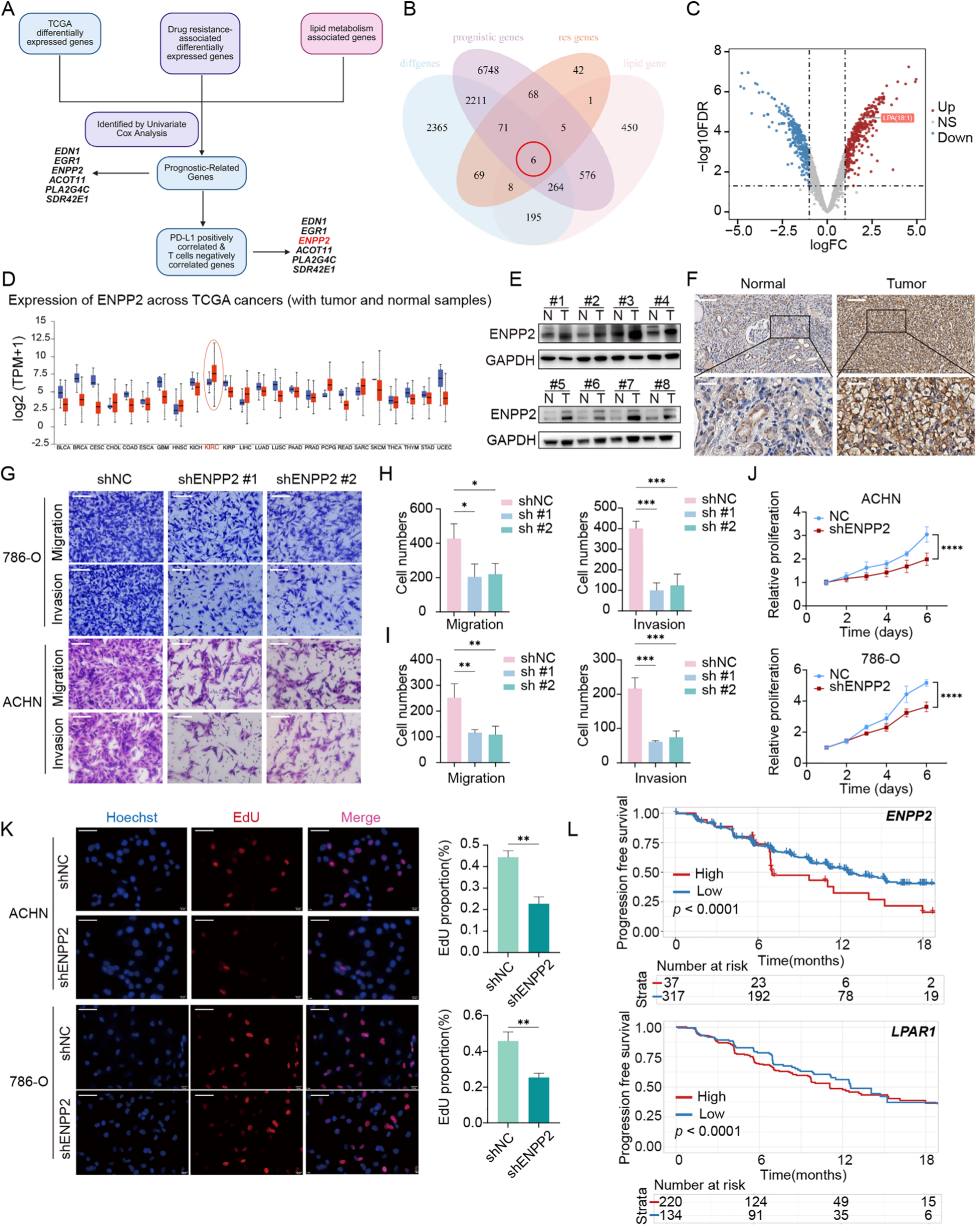
ENPP2 is highly expressed in RCC and correlates with malignant phenotypes. (A) Schematic workflow for screening lipid‐metabolism–related genes involved in TKI resistance. (B) Venn diagram showing the overlap among differentially expressed genes from the TCGA cohort (diffgenes), TKI‐resistant cells (res genes), prognostic genes from TCGA (prognostic genes), and lipid‐metabolism–related genes (lipid genes). (C) Volcano plot of differential lipid species identified by targeted lipidomics in TKI‐resistant versus sensitive RCC cells. Up, upregulation; NS, not significant; Down, downregulation. (D) Pan‐cancer mRNA expression of ENPP2 in tumor versus adjacent normal tissues (TCGA). (E, F) ENPP2 upregulation in RCC patient tissues validated by Western blotting (E) and representative immunohistochemistry (F). Scale bars: 100 µm. (G‐I) Representative images (G) and quantification of transwell migration and invasion assays for 786‐O (H) and ACHN (I) cells. Cells (50 000/well) were seeded into chambers with or without Matrigel coating and fixed after 12 h (migration) or 24 h (invasion). Scale bars: 100 µm. (J) Proliferation curves of ACHN and 786‐O cells (1000 cells/well) measured by CCK‐8 assay at the indicated time points. (K) Representative images and quantification of EdU incorporation assays. Scale bars: 20 µm. (L) Kaplan‐Meier survival curves for ENPP2 (high vs. low) and LPAR1 (high vs. low) expression in Javelin101 cohorts. The number of patients at risk at the indicated time points is shown below the Kaplan–Meier curves. All quantitative in vitro data are presented as mean ± SD from *n* = 3 independent experiments. Statistical significance was determined using an unpaired two‐tailed Student's *t*‐test (K), one‐way ANOVA (H, I), or two‐way ANOVA (J), followed by Tukey's post hoc test. ns = not significant, *p < 0.05, **p < 0.01, ***p < 0.001, ****p < 0.0001.

Previous studies implicated autotaxin (ATX), the protein encoded by ENPP2, in tumor progression through its enzymatic production of lysophosphatidic acid (LPA) [23]. To investigate the metabolic alterations associated with this axis in RCC, we conducted targeted lipid metabolomics on TKI‐resistant and sensitive cell lines, revealing significant accumulation of LPA in resistant populations compared with their sensitive counterparts (Figure 1C). Detailed profiling of individual LPA species revealed a specific compositional remodeling of the intracellular lipid pool in cabozantinib‐resistant cells, specifically, the highly bioactive unsaturated species, LPA (18:1), exhibited a profound and statistically significant accumulation. (Figure S1E).

A pan‐cancer analysis revealed ENPP2 as strikingly upregulated in clear cell renal cell carcinoma (KIRC) compared to its generally low expression across other malignancies (Figure 1D). Multi‐omics validation using TCGA transcriptomic data and CPTAC proteomic profiles confirmed elevated ENPP2 expression at both mRNA and protein levels in KIRC tumors (Figure S1F,G). Concordantly, western blotting and immunohistochemistry (IHC) of patient‐derived RCC tissues corroborated tumor‐specific ENPP2 overexpression (Figure 1E,F). To determine whether ENPP2 functionally contributes to renal cancer malignancy, we generated stably knocked‐down and overexpressed ENPP2 cell lines in 786‐O and ACHN renal cancer cell lines (Figure S1H). Transwell assays demonstrated that ENPP2 knockdown significantly reduced the migratory and invasive capacities of renal cancer cells (Figure 1G–I). CCK‐8 assays and EdU incorporation assays further confirmed that ENPP2 depletion impaired renal cancer cell proliferation (Figure 1J,K), whereas ENPP2 overexpression exhibited the opposite effects (Figure S1I–L).

We next evaluated the clinical relevance of the ENPP2–LPA receptor (LPAR) axis. Survival analysis of the JAVELIN Renal 101 combination therapy cohort (avelumab plus axitinib) revealed that elevated ENPP2 and LPAR1 expression correlated with poorer clinical outcomes (Figure 1L). To further validate the prognostic significance of this axis across broader clinical populations, we analyzed the TCGA and CPTAC ccRCC datasets (Figure S2A–E). High LPAR1 mRNA expression was significantly associated with poor overall survival in both the TCGA and CPTAC transcriptomic cohorts (*p* < 0.0001). While ENPP2 mRNA levels in these bulk cohorts paradoxically correlated with favorable survival, analysis of the CPTAC global proteomic dataset revealed that elevated ENPP2 protein expression was significantly associated with poor overall survival (*p* < 0.0001) (Figure S2E). Collectively, these findings identify the ENPP2–LPA axis as a potential driver of TKI resistance and immune escape in RCC, prompting us to further investigate its underlying molecular mechanisms.

### 3.2 ENPP2 Modulates TKI Sensitivity in Renal Cancer Cells Both In Vitro and In Vivo

Given the elevated expression of ENPP2 and its metabolite LPA in TKI‐resistant renal cancer cell lines, we further investigated whether ENPP2 influences the sensitivity of renal cancer cells to TKI drugs. Following the in vivo establishment of cabozantinib‐resistant 786‐O and ACHN cell lines (designated as 786‐R and ACHN‐R, respectively) (Figure 2A), western blotting and qRT‐PCR confirmed marked upregulation of ENPP2 in these resistant cells compared to their drug‐sensitive parental counterparts (Figure 2B,C). The enhanced drug tolerance of these resistant cell lines was validated through TUNEL, CCK‐8 and colony formation assays (Figure S3A–D). Subsequent ENPP2 knockdown in both drug‐resistant (786‐R and ACHN‐R) and parental cell lines significantly enhanced cabozantinib sensitivity (Figure 2D, Figure S3E), whereas ENPP2 overexpression conferred cabozantinib resistance (Figure 2E). Consistently, colony formation and flow cytometric apoptosis assays further demonstrated that ENPP2 depletion reduced tumor cell proliferation and enhanced apoptosis under cabozantinib treatment (Figure 2F–H, Figure S3F). To ensure these functional phenotypes were not limited to acquired resistance models, we profiled endogenous ENPP2 expression across a panel of human RCC cell lines and identified OSRC2 as exhibiting the highest baseline levels (Figure S4A). Subsequent shRNA‐mediated silencing of ENPP2 in OSRC2 cells (Figure S4B) profoundly sensitized them to cabozantinib, as demonstrated by decreased cell viability, increased apoptosis rates, and diminished colony‐forming capacity (Figure S4C–E). For in vivo validation, xenograft models in BALB/c nude mice revealed that ENPP2 depletion alone achieved tumor growth inhibition comparable to cabozantinib monotherapy. Strikingly, combining ENPP2 knockdown with cabozantinib produced more potent antitumor effects, as quantified by significantly reduced tumor volumes and weights (Figure 2I–L). Collectively, these findings establish that ENPP2 functionally modulates cabozantinib sensitivity both in vitro and in vivo.

**FIGURE 2:**
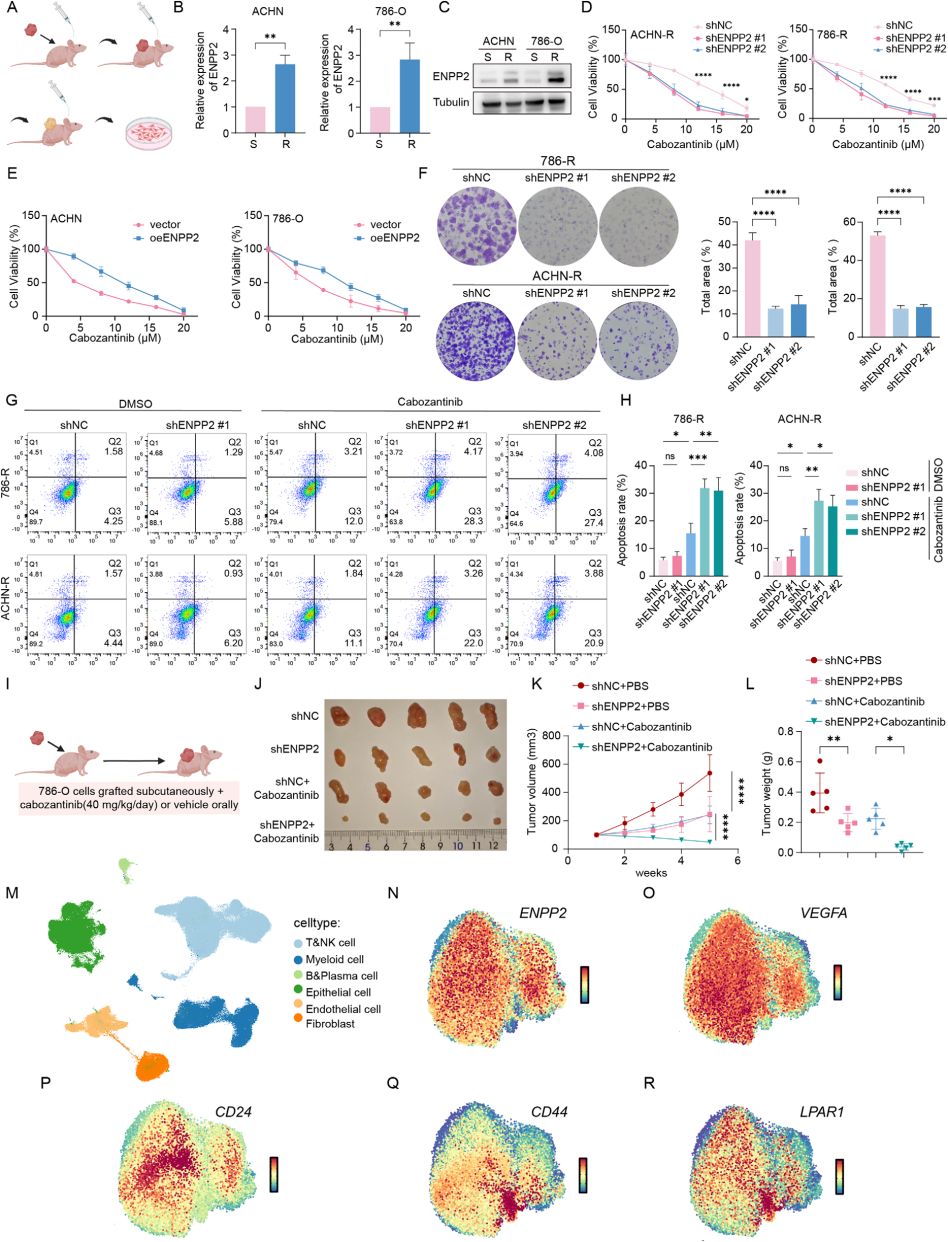
ENPP2 modulates TKI sensitivity in renal cancer cells both in vitro and in vivo. (A) Schematic workflow for generating cabozantinib‐resistant RCC cell lines in vivo. (B, C) qRT‐PCR analysis (B) and immunoblotting (C) confirming the upregulation of ENPP2 mRNA and protein levels in cabozantinib‐resistant (R) ACHN and 786‐O sublines compared to their drug‐sensitive parental (S) counterparts. (D, E) CCK‐8 viability assays of resistant cells (ACHN‐R and 786‐R) transduced with control shRNA (shNC) or independent ENPP2 shRNAs (shENPP2 #1 and #2) (D), and parental cells (ACHN and 786‐O) overexpressing ENPP2 (oeENPP2) or empty vector (E) across a cabozantinib concentration gradient. (F) Representative images of colony formation assays of resistant cells after ENPP2 knockdown treated with 5 µM cabozantinib for 7–14 days, the statistical results are presented in right panel. (G, H) Representative flow‐cytometry plots (G) and quantification of apoptotic cells (H) in 786‐R and ACHN‐R cells expressing shNC or shENPP2 and treated with 5 µM cabozantinib or DMSO for 48 h. (I) Schematic of the in vivo therapeutic regimen: 786‐O cells with stable shNC or shENPP2 were implanted subcutaneously into BALB/c nude mice, followed by oral administration of cabozantinib (40 mg/kg/day) or vehicle (PBS). (J) Representative images of excised xenograft tumors from the indicated groups (*n* = 5 mice per group). (K) Tumor growth curves; tumor volume was calculated as 0.5 × length × width^2^. (L) Final tumor weights at the study endpoint. (M) UMAP visualization of single‐cell RNA‐sequencing data identifying six major cell populations based on canonical marker genes. (N, O) Feature plots depicting ENPP2 and VEGFA expression patterns in the tumor microenvironment. (P–R) Feature plots of CD24, CD44, and LPAR1 expression, illustrating the specific enrichment of LPAR1 within the CD44^+^CD24^−^ epithelial cell subset. Quantitative in vitro data are presented as mean ± SD from *n* = 3 independent experiments. In vivo data are presented as mean ± SD with *n* = 5 mice per group. Statistical significance was determined using an unpaired two‐tailed Student's t‐test (B), one‐way ANOVA (F, H, L), or two‐way ANOVA (D, E, K), followed by Tukey's post hoc test. ns = not significant, *p < 0.05, **p < 0.01, ***p < 0.001, ****p < 0.0001.

To further elucidate the role of ENPP2 within the tumor microenvironment of RCC at the single‐cell resolution, we integrated single‐cell RNA sequencing (scRNA‐seq) data from public datasets. Following rigorous quality control, a total of 355188 high‐confidence cells were clustered into six major populations based on canonical marker genes (Figure 2M, Figure S4F). Within the malignant epithelial cluster, a high correlation between ENPP2 and VEGFA expression patterns was observed (Figure 2N,O), and ENPP2 expression was positively correlated with hypoxia and angiogenesis pathways (Figure S4G). Interestingly, the CD44^+^CD24^−^ subpopulation, known to be enriched in cancer stem cell (CSC) characteristics, displayed elevated LPAR1 expression (Figure 2P–R) [38, 39]. Given that alternative activation of cancer stemness and angiogenic programs has been implicated in resistance to targeted therapies [40, 41], these results provided a plausible mechanistic explanation for ENPP2‐driven therapeutic resistance in RCC.

### 3.3 ENPP2 Catalytic Activity and Its Metabolite LPA Mediate TKI Resistance in Renal Cancer Cells

Given the established role of ENPP2 in catalyzing LPA production and initiating downstream LPA receptor signaling [19, 42], we next investigated whether ENPP2‐mediated TKI resistance in renal cancer cells depends on its catalytic activity and metabolite LPA. We employed two ENPP2 inhibitors, GLPG1690 and HA130, to suppress ENPP2 enzymatic activity pharmacologically. CCK‐8 assays demonstrated that pharmacological inhibition of ENPP2 activity significantly restored cabozantinib sensitivity in resistant RCC cells (Figure 3A). Consistent sensitization was observed in colony formation and apoptosis assays, with both inhibitors effectively abrogating the resistant phenotype (Figure 3B–E). Correspondingly, ELISA quantification revealed a substantial decrease in secreted LPA concentrations within the culture supernatants of ENPP2‐knockdown renal cancer cells (Figure 3F). Importantly, supplementation with exogenous LPA rescued cabozantinib sensitivity in drug‐naïve cells (Figure 3G–I) and reversed the sensitizing effects induced by ENPP2 knockdown (Figure 3J,K). To definitively link enzymatic activity to resistance, we generated an enzymatically dead ENPP2 mutant by mutating the 210th nucleotide from thymine (T) to adenine (A) in the active site (T210A), which abolishes catalytic function as previously reported [25]. Immunoblot analysis verified equivalent protein expression levels between the wild‐type and T210A mutant constructs (Figure S4H). While wild‐type ENPP2 overexpression conferred cabozantinib resistance, the T210A mutant failed to alter drug response (Figure 3L,M), demonstrating that the enzymatic activity of ENPP2 is essential for its resistance‐promoting effects in RCC.

**FIGURE 3:**
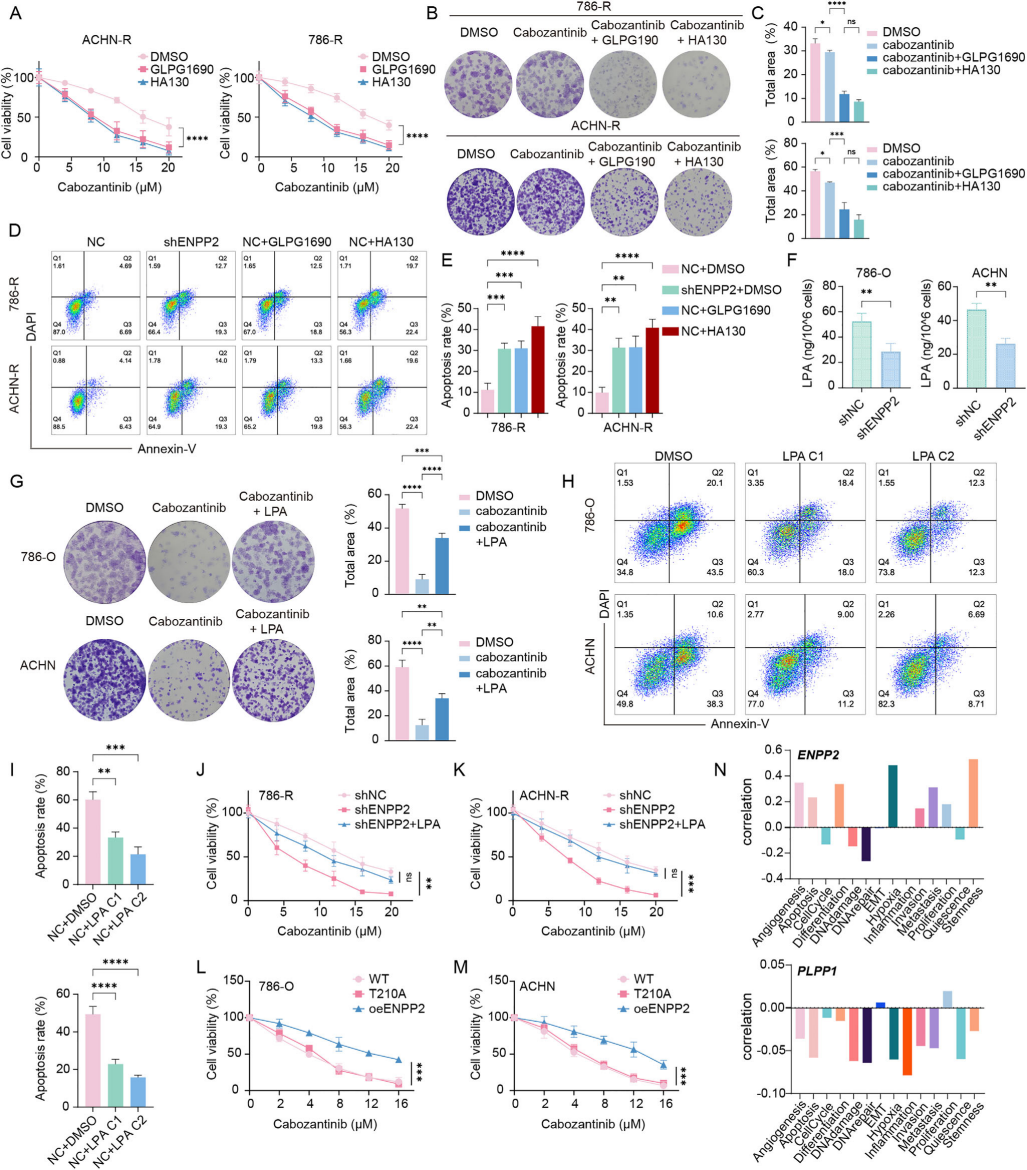
ENPP2 drives cabozantinib resistance through its enzymatic activity and LPA biosynthesis. (A) CCK‐8 viability assays of ACHN‐R and 786‐R cells treated with 10 µM GLPG1690, 10 µM HA130, or DMSO control, across a concentration gradient of cabozantinib for 48 h. (B, C) Representative images (B) and total area quantification (C) of colony formation assays for resistant cells treated with 5 µM cabozantinib in the presence of 10 µM GLPG1690, 10 µM HA130, or DMSO. (D, E) Representative Annexin V/DAPI flow cytometry plots (D) and apoptosis rate quantification (E) of 786‐R and ACHN‐R cells transduced with control shRNA (NC) or shENPP2, and treated with 5 µM cabozantinib plus 10 µM GLPG1690, 10 µM HA130, or DMSO for 48 h. (F) ELISA quantification of secreted LPA levels in cell culture supernatants from shNC and shENPP2 RCC cells cultured in low‐serum medium (0.5% FBS) for 6 h. (G) Colony formation assays of parental 786‐O and ACHN cells treated with DMSO control, 5 µM cabozantinib alone, or 5 µM cabozantinib combined with 10 µM exogenous LPA. (H, I) Flow cytometric apoptosis analysis (H) and quantification (I) of parental cells treated with 5 µM cabozantinib and co‐treated with DMSO or exogenous LPA (C1 = 10 µM; C2 = 20 µM) for 48 h. (J, K) CCK‐8 viability assays of 786‐R and ACHN‐R cells expressing shNC or shENPP2, treated with cabozantinib at the indicated concentrations with or without 20 µM LPA. (L, M) CCK‐8 assays demonstrating that overexpression of wild‐type ENPP2 (oeENPP2) enhanced TKI resistance in sensitive cell lines, while no such effect was observed in cells that overexpressed the enzymatically inactive T210A mutant. (N) Correlations between ENPP2 (top) or PLPP1 (bottom) expression and selected functional states of tumor cells obtained from the CancerSea database. All quantitative in vitro data are presented as mean ± SD from n = 3 independent experiments. Statistical significance was determined using an unpaired two‐tailed Student's t‐test (F), one‐way ANOVA (C, E, G, I), or two‐way ANOVA (A, J, K, L, M), followed by Tukey's post hoc test. ns = not significant, *p < 0.05, **p < 0.01, ***p < 0.001, ****p < 0.0001.

To contextualize these metabolic findings within broader tumor functional states, we analyzed single‐cell datasets from CancerSea. ENPP2 expression positively correlated with functional signatures of angiogenesis, hypoxia, metastasis, and stemness. Conversely, the expression of PLPP1, a major LPA‐catabolizing enzyme, exhibited a perfectly inverse correlation pattern across these functional states (Figure 3N).

### 3.4 ENPP2 Mediates TKI Resistance in Renal Cell Carcinoma Through AKT/mTOR Pathway

To elucidate the specific pathways through which ENPP2 influences TKI resistance in renal cancer, we performed transcriptomic sequencing in 786‐O cells following ENPP2 knockdown and conducted pathway enrichment analysis of differentially expressed genes. Gene Ontology (GO) enrichment analysis indicated a significant association with T cell‐mediated immune responses (Figure 4A), while Gene Set Enrichment Analysis (GSEA) analysis demonstrated a marked downregulation of the AKT/mTOR signaling axis upon ENPP2 silencing (Figure 4B). Western blotting confirmed reduced phosphorylation of AKT (Ser473) and mTOR (Ser2448) in ENPP2‐depleted cells (Figure 4C), whereas ENPP2 overexpression conversely enhanced their activation (Figure 4D). Furthermore, pharmacological inhibition of ENPP2 abrogated this phosphorylation cascade in ENPP2‐overexpressing cells, an effect that was subsequently reversed by exogenous LPA supplementation (Figure 4E). To eliminate the endogenous influence of ENPP2 entirely, we generated ENPP2 knockout (KO) RCC lines (786‐O and ACHN) via CRISPR‐Cas9, followed by reconstitution with either wild‐type ENPP2 (oeENPP2) or catalytically inactive T210A mutant ENPP2. Immunoblotting showed that only the wild‐type ENPP2‐transfected group exhibited elevated phosphorylation of AKT and mTOR (Figure 4F), confirming that this signaling cascade is strictly dependent on ENPP2 enzymatic activity.

**FIGURE 4:**
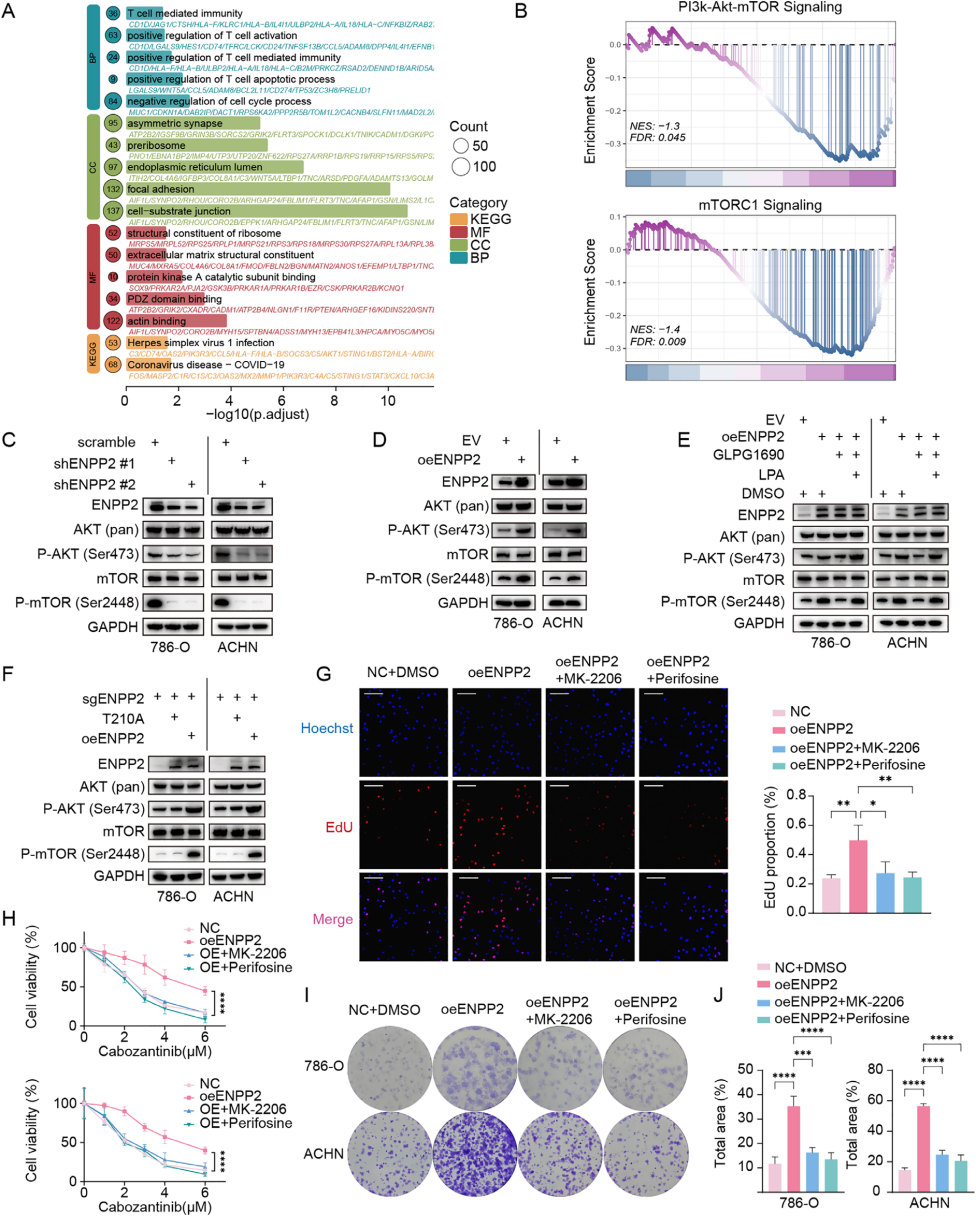
ENPP2 mediates TKI resistance in RCC via catalytic activity‐dependent activation of the AKT/mTOR pathway. (A) GO and KEGG enrichment analysis results of RNA‐seq data from ENPP2‐knockdown 786‐O cells. (B) GSEA plots showing negative enrichment of PI3K–AKT–mTOR and mTORC1 signaling signatures in ENPP2‐depleted cells. (C, D) Immunoblot analysis of ENPP2, total AKT, phospho‐AKT (Ser473), total mTOR, and phospho‐mTOR (Ser2448) in 786‐O and ACHN cells following ENPP2 knockdown with two independent shRNAs (C) or ectopic ENPP2 overexpression (D). (E) Immunoblot analysis of AKT/mTOR cascade activation in oeENPP2‐expressing 786‐O and ACHN cells treated with DMSO control, the ENPP2 inhibitor GLPG1690 (10 µM), or GLPG1690 combined with exogenous LPA (20 µM) for 48 h. (F) Immunoblot analysis of the specified cascade proteins in ENPP2‐knockout (sgENPP2) 786‐O and ACHN cells reconstituted with either wild‐type ENPP2 (oeENPP2) or the catalytically inactive T210A mutant. (G) Representative fluorescence images and quantification of EdU incorporation assays in RCC cells overexpressing ENPP2, with or without co‐treatment with the AKT inhibitors MK‐2206 (10 µM) or perifosine (10 µM) for 6 h. Scale bars: 20 µm. (H) CCK‐8 assays demonstrating that AKT pharmacologically inhibition (MK‐2206 or Perifosine) attenuates ENPP2‐induced cabozantinib resistance in 786‐O and ACHN cells. (I, J) Representative images (I) and total area quantification (J) of colony formation assays for 786‐O and ACHN cells treated as in (G) under cabozantinib pressure. All quantitative in vitro data are presented as mean ± SD from *n* = 3 independent experiments. Statistical significance was determined using one‐way ANOVA (G, J), or two‐way ANOVA (H), followed by Tukey's post hoc test. *p < 0.05, **p < 0.01, ***p < 0.001, ****p < 0.0001.

To determine the functional requirement of the AKT/mTOR axis in ENPP2‐mediated TKI resistance, we employed two selective AKT inhibitors, MK‐2206 and Perifosine. EdU assays demonstrated that pharmacological AKT inhibition effectively counteracted the hyperproliferative phenotype induced by ENPP2 overexpression (Figure 4G). Moreover, CCK‐8 and colony formation assaysrevealed that AKT inhibition significantly attenuated the ENPP2 overexpression‐induced cabozantinib resistance enhancement in RCC cells (Figure 4H–J). Collectively, these data demonstrate that ENPP2 promotes RCC proliferation and TKI resistance via a catalytic activity‐dependent activation of the AKT/mTOR signaling pathway.

### 3.5 ENPP2 Drives PD‐L1 Expression and T‐Cell Dysfunction via the Catalytic Activity–Dependent TBK1/IRF3 Axis

Building on the observed correlation between ENPP2 and PD‐L1 expression from transcriptomic screening, coupled with GO analysis implicating ENPP2 in T cell‐mediated immunity, we performed GSEA to identify immune‐related pathways modulated by ENPP2. ENPP2 knockdown significantly attenuated interferon‐α/‐γ response pathways (Figure 5A), which are mechanistically linked to TBK1/IRF3 signaling and subsequent IRF3‐driven PD‐L1 transcriptional activation. Flow cytometric analysis confirmed that ENPP2 depletion reduced, whereas ENPP2 overexpression enhanced, PD‐L1 surface expression on renal cancer cells (Figure 5B, Figure S5A,B). Western blot experiments further validated the suppressive effect of ENPP2 depletion on PD‐L1 expression in renal cancer cells, accompanied by decreased phosphorylation of TBK1 and IRF3; conversely, ENPP2 overexpression produced opposing effects (Figure 5C, Figure S5C). Treatment with the ENPP2 inhibitor GLPG1690 effectively blocked the activation of TBK1 and IRF3 induced by ENPP2 overexpression, while exogenous LPA supplementation rescued their activation (Figure 5D). Notably, although wild‐type ENPP2 overexpression significantly activated TBK1 and IRF3, the catalytically inactive T210A mutant failed to elicit this effect (Figure 5E). Collectively, these findings demonstrate that ENPP2 regulates PD‐L1 expression through catalytic activity–dependent modulation of TBK1/IRF3 phosphorylation.

**FIGURE 5:**
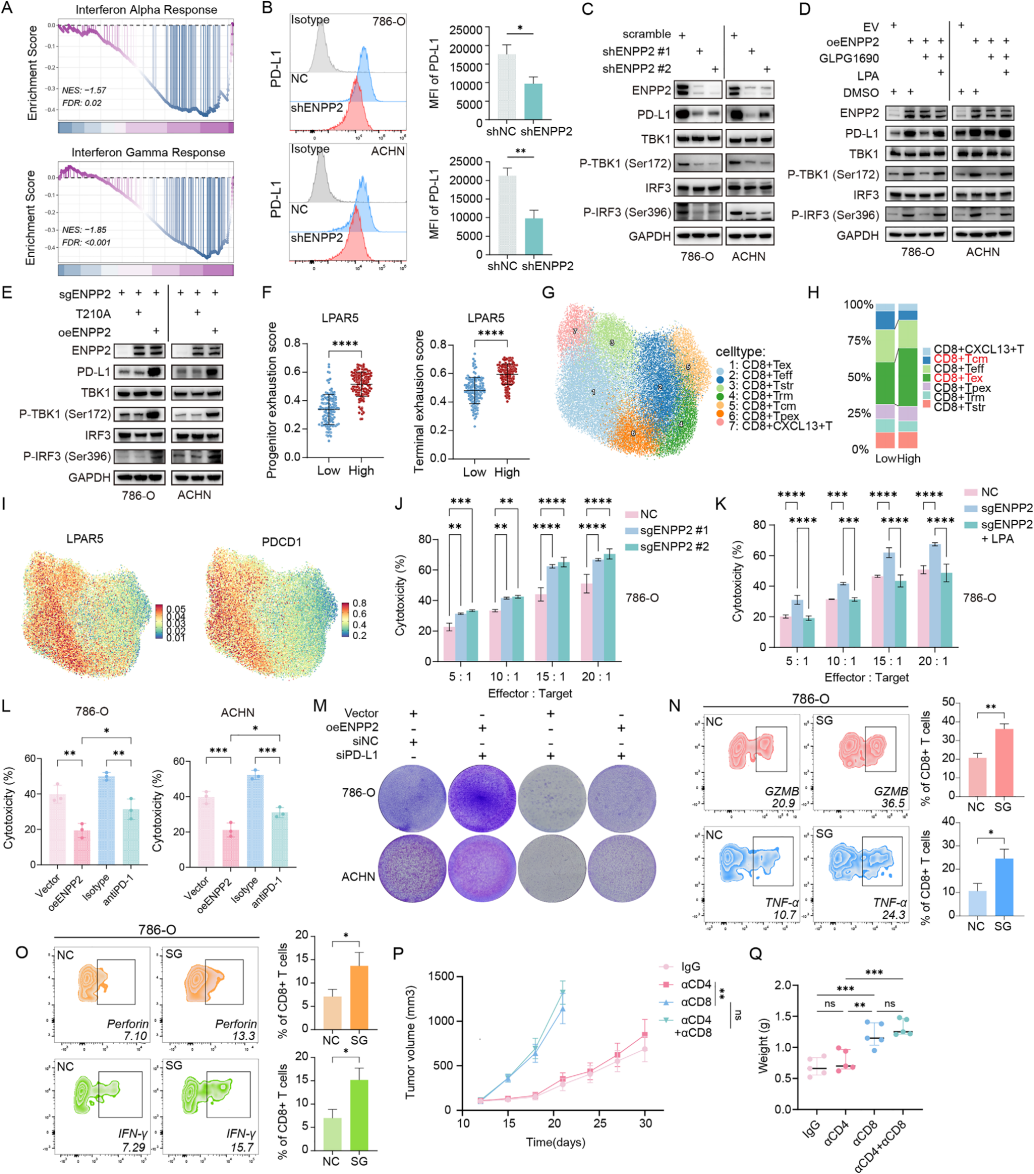
ENPP2 drives immune evasion via the TBK1/IRF3/PD‐L1 axis and suppresses CD8^+^ T‐cell effector functions in renal cancer cells. (A) GSEA plots showing negative enrichment of interferon‐α and interferon‐γ response signatures following ENPP2 knockdown. (B) Representative flow cytometry histograms and quantification of median fluorescence intensity (MFI) for surface PD‐L1 expression in 786‐O and ACHN cells transduced with control shRNA (shNC) or shENPP2. (C) Immunoblot analysis of ENPP2, PD‐L1, total TBK1, phospho‐TBK1 (Ser172), total IRF3, and phospho‐IRF3 (Ser396) in 786‐O and ACHN cells following ENPP2 knockdown. (D) Immunoblot analysis of the TBK1/IRF3/PD‐L1 axis in ENPP2‐overexpressing 786‐O and ACHN cells treated with 10 µM GLPG1690, 10 µM GLPG1690 plus 20 µM LPA, or DMSO control for 48 h. (E) Immunoblot analysis of the specified cascade proteins in ENPP2‐knockout (sgENPP2) cells reconstituted with either wild‐type ENPP2 (oeENPP2) or the catalytically inactive T210A mutant. (F) Progenitor and terminal exhaustion scores of CD8+ T cells stratified by LPAR5 expression level. (G) UMAP visualization of T‐cell subclusters identified by single‐cell RNA‐seq (Tex, exhausted T cells; Teff, effector T cells; Tstr, stress‐response T cells; Trm, tissue‐resident memory T cells; Tcm, central memory T cells; Tpex, progenitor‐exhausted T cells; CXCL13+ T, CD8+CXCL13+ T cells). (H) Proportions of the indicated CD8+ T‐cell subpopulations in tumors with low versus high ENPP2 expression. (I) Feature plots showing co‐localization of LPAR5 and PDCD1 (PD‐1) expression in tumor‐infiltrating T cells. (J, K) LDH‐release cytotoxicity assays of primary human T cells co‐cultured with control (NC) or ENPP2‐knockout (sgENPP2) 786‐O cells across varying effector‐to‐target (E:T) ratios (J), and the corresponding rescue effect of exogenous LPA supplementation (K). (L, M) LDH‐release cytotoxicity assay (L) and representative crystal violet staining (M) of oeENPP2 RCC cells co‐cultured with T cells, demonstrating the restorative effects of pharmacological anti‐PD‐1 antibody treatment or genetic PD‐L1 knockdown (siPD‐L1) on T‐cell‐mediated killing. (N, O) Representative flow cytometry plots and quantification of Granzyme B (GZMB) and TNF‐α (N), as well as Perforin and IFN‐γ (O) in CD8^+^ T cells after co‐culture with control (NC) or ENPP2‐knockout (SG) 786‐O cells. (P, Q) In vivo antibody‐mediated CD4^+^ and CD8^+^ T‐cell depletion in an immunocompetent syngeneic RCC model. Tumor growth kinetics (P) and final endpoint tumor weights (Q) of the indicated depletion cohorts (αCD4, αCD8, or αCD4+αCD8) compared to the IgG control. Quantitative in vitro data are presented as mean ± SD from *n* = 3 independent experiments. In vivo data are presented as mean ± SD with *n* = 5 mice per group. Statistical significance was determined using an unpaired two‐tailed Student's *t*‐test (B, F, N, O), one‐way ANOVA (L, Q), or two‐way ANOVA (J, K, P), followed by Tukey's post hoc test. ns = not significant, *p < 0.05, **p < 0.01, ***p < 0.001, ****p < 0.0001.

To evaluate the impact of ENPP2 on the tumor immune microenvironment, we applied the TcellSI scoring tool to bulk transcriptomic data [43]. In the ENPP2‐high expression group, T cell proliferation scores were lower (Figure S5D), whereas the LPAR5‐high expression group exhibited increased exhaustion scores (Figure 5F). We further extracted CD8+ T cells from the previously mentioned single‐cell dataset and classified them into seven subpopulations based on marker genes (Figure 5G, Figure S5E). Cell composition profiling revealed that ENPP2‐high tumors contained fewer circulating memory CD8+ T cells and an enrichment of exhausted CD8+ T cells (Figure 5H). Moreover, LPAR5 expression positively correlated with inhibitory exhaustion markers including PD‐1, LAG‐3, and CTLA‐4 (Figure 5I, Figure S5F,G).

To directly assess how ENPP2 in tumor cells influences T‐cell cytotoxic activity, we conducted co‐culture assays using primary human T cells and either wild‐type or ENPP2‐knockout (KO) 786‐O and ACHN renal cancer cells at varying effector‐to‐target ratios. LDH cytotoxicity assays and colony formation assays demonstrated that T cells co‐cultured with ENPP2‐KO tumor cells exhibited significantly enhanced cytotoxic activity compared to controls (Figure 5J, Figure S5H,I), an effect that was abrogated by exogenous LPA supplementation (Figure 5K, Figure S5J,K). To further elucidate the role of PD‐L1 in ENPP2‐induced immune suppression, we performed additional co‐culture assays using ENPP2‐overexpressing renal cancer cells in the presence of isotype control antibody, anti–PD‐1 antibody, or PD‐L1 knockdown. Genetic or pharmacological blockade of the PD‐1/PD‐L1 axis substantially diminished the survival advantage conferred by ENPP2 overexpression, yet a residual suppression of T‐cell cytotoxicity persisted (Figure 5L,M). Flow cytometric analysis further demonstrated that T cells co‐cultured with ENPP2‐knockout 786‐O cells secreted significantly higher levels of cytotoxic granules (GZMB and Perforin) and cytokines (IFN‐γ and TNF‐α) compared to control groups (Figure 5N,O). Similar trends were obtained in T cells co‐cultured with ENPP2‐knockout ACHN cells (Figure S5L,M).

To establish the in vivo requirement of T‐cell for ENPP2‐mediated immune evasion, we performed antibody‐mediated cell depletion in an immunocompetent syngeneic RCC model. While neutralizing CD4^+^ T cells (αCD4) did not alter tumor growth compared to the IgG control, specific depletion of CD8^+^ T cells (αCD8), either alone or concurrently with CD4^+^ depletion, drastically accelerated tumor growth (Figure 5P) and significantly increased endpoint tumor burden (Figure 5Q). Together, these in vitro and in vivo results demonstrate that ENPP2 orchestrates tumor immune evasion and suppresses CD8^+^ T‐cell effector functions primarily by driving PD‐L1 expression through the TBK1/IRF3 signaling axis.

### 3.6 LPAR1 Specifically Mediates ENPP2‐Driven TKI Resistance by Activating a TBK1‐AKT/mTOR Cascade

To investigate the specific lysophosphatidic acid receptor (LPAR) mediating ENPP2‐induced TKI resistance in RCC, we first examined the baseline expression profiles of LPAR1–6 in 786‐O and ACHN cell lines. LPAR1 was found to be predominantly expressed compared to other receptors (Figure 6A). To confirm LPAR1 as the primary functional receptor for ENPP2 signaling, we employed both pharmacological blockade and genetic depletion. Treatment with AM966, a specific LPAR1 antagonist, profoundly abrogated the ENPP2‐overexpression‐induced phosphorylation of downstream targets, including TBK1, IRF3, AKT, and mTOR (Figure 6B). Conversely, knockdown of LPAR2 failed to suppress this signaling cascade (Figure 6C). Functionally, LPAR1 silencing significantly sensitized RCC cells to cabozantinib‐induced apoptosis and viability inhibition in vitro (Figure 6D,F,G, Figure S6A). Importantly, parallel knockdown of LPAR2 failed to alter cabozantinib sensitivity (Figure 6E, Figure S6A), indicating that ENPP2‐mediated drug resistance is LPAR1‐dependent. To validate these findings in vivo, we established a xenograft model using 786‐O cells. In line with the in vitro data, tumors with stable LPAR1 knockdown exhibited a significantly decelerated growth rate under cabozantinib treatment, as tracked by bioluminescence imaging (Figure 6H,I), supporting the indispensable role of LPAR1 in conferring drug resistance.

**FIGURE 6:**
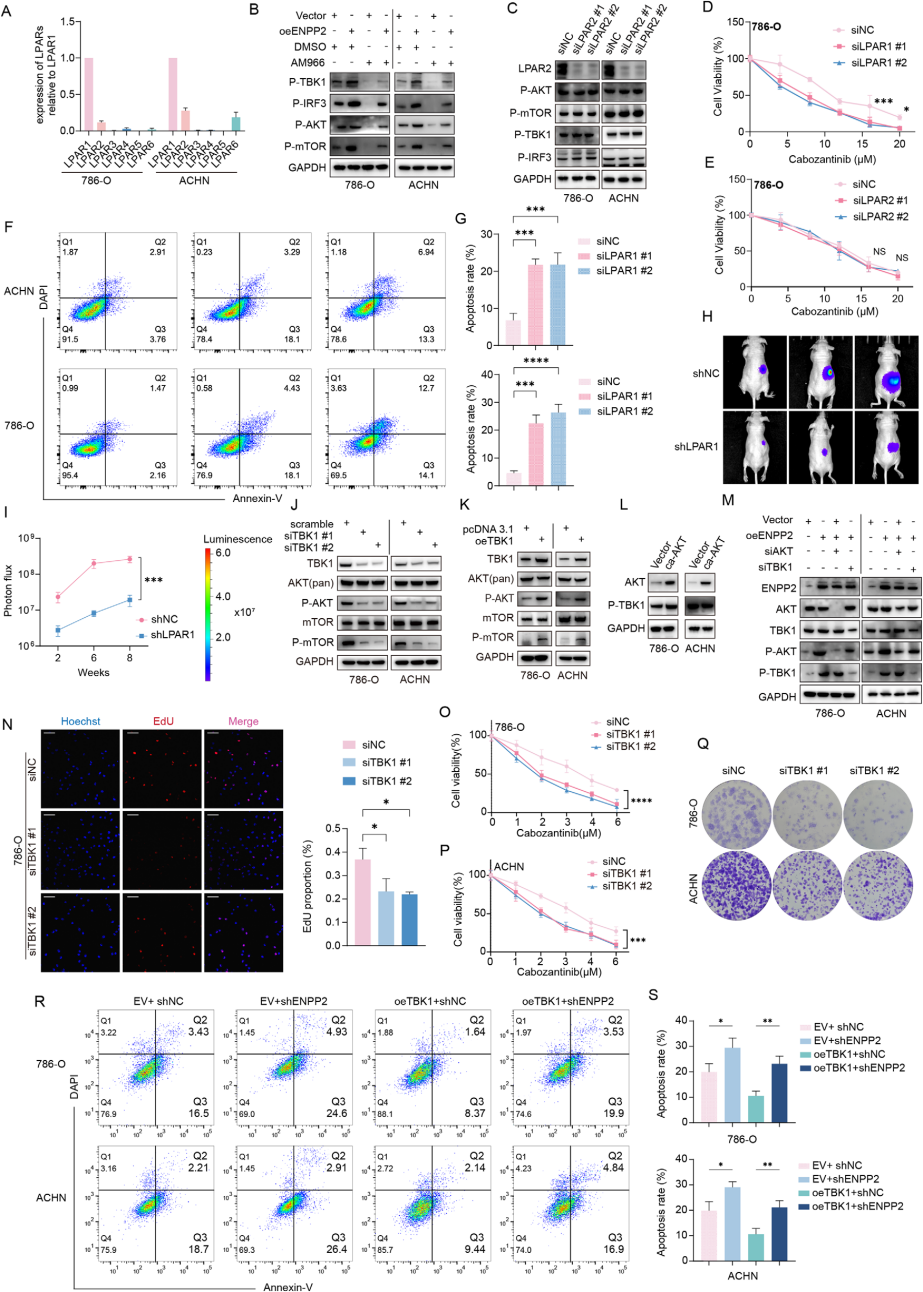
LPAR1 mediates ENPP2‐driven TKI resistance by activating a TBK1‐AKT/mTOR cascade. (A) Baseline mRNA expression profiles of LPAR1–6 in 786‐O and ACHN cell lines, determined by qRT‐PCR. (B) Immunoblot analysis of downstream cascade activation (TBK1, IRF3, AKT, and mTOR) in ENPP2‐overexpressing RCC cells treated with DMSO or the specific LPAR1 antagonist AM966. (C) Immunoblot analysis confirming that genetic depletion of LPAR2 (siLPAR2 #1 and #2) fails to abrogate ENPP2‐induced downstream signaling. (D–G) In vitro viability evaluated by CCK‐8 assays across cabozantinib concentration gradients (D, E) and flow cytometric apoptosis analysis (F, G) of RCC cells following LPAR1 silencing (D, F, G) or LPAR2 silencing (E), demonstrating that cabozantinib sensitization is LPAR1‐dependent. (H, I) Representative in vivo bioluminescence images (H) and quantification of tumor growth kinetics (I) for 786‐O xenografts harboring stable LPAR1 knockdown under cabozantinib treatment (n = 5 mice per group). (J, K) Immunoblot analysis of AKT and mTOR phosphorylation following TBK1 knockdown (J) or ectopic TBK1 overexpression (K). (L) Immunoblot analysis of TBK1 and AKT phosphorylation in RCC cells overexpressing a constitutively active AKT mutant (ca‐AKT). (M) Immunoblot analysis of TBK1 and AKT phosphorylation in ENPP2‐overexpressing cells following TBK1 or AKT silencing. (N) EdU incorporation assays in RCC cells following TBK1 depletion. (O, P) CCK‐8 cell viability assays of TBK1‐depleted RCC cells across a cabozantinib concentration gradient. (Q) Colony formation assays of TBK1‐depleted RCC cells under cabozantinib treatment. (R, S) Representative flow cytometry plots (R) and apoptosis rate quantification (S) demonstrating that ectopic expression of TBK1 (oeTBK1) effectively rescues cell survival in ENPP2‐depleted cells under cabozantinib treatment. Data are presented as mean ± SD from *n* = 3 independent in vitro experiments or *n* = 5 mice per group. Statistical significance was determined using a one‐way ANOVA (G, N, S), or two‐way ANOVA (D, E, I, O, P), followed by Tukey's post hoc test. ns = not significant, *p < 0.05, **p < 0.01, ***p < 0.001, ****p < 0.0001.

We next delineated the hierarchical relationship between TBK1 and the AKT/mTOR pathway within this specific signaling axis. Western blot analysis revealed that TBK1 knockdown markedly reduced the phosphorylation of AKT and mTOR (Figure 6J), whereas ectopic TBK1 expression produced the reciprocal activation (Figure 6K). To definitively establish the unidirectional flow of this cascade, we performed an epistatic analysis. Overexpression of a constitutively active AKT mutant (ca‐AKT) did not alter the upstream phosphorylation of TBK1 (Figure 6L). Furthermore, while ENPP2 overexpression concurrently hyperactivated both TBK1 and AKT, co‐silencing of TBK1 effectively abolished the phosphorylation of both kinases. In contrast, co‐silencing of AKT only abolished p‐AKT levels while leaving the upstream p‐TBK1 intact (Figure 6M). These genetic rescue data unequivocally position TBK1 upstream of the AKT/mTOR pathway within the ENPP2 signaling axis.

Phenotypically, TBK1 depletion suppressed the proliferative capacity of RCC cells, as evidenced by EdU incorporation assays (Figure 6N), and significantly enhanced cellular sensitivity to cabozantinib, demonstrated by reduced cell viability (Figure 6O,P), diminished colony formation (Figure 6Q, Figure S6B), and increased apoptosis rates (Figure S6C). Finally, to confirm that this specific cascade is responsible for the resistant phenotype, we conducted flow cytometric apoptosis rescue assays. While ENPP2 knockdown robustly potentiated cabozantinib‐induced apoptosis, ectopic expression of TBK1 in these ENPP2‐depleted cells effectively reinstated the drug‐resistant phenotype, significantly rescuing cell survival (Figure 6R,S). Collectively, these data demonstrate that ENPP2 confers TKI resistance in RCC through specific engagement with LPAR1 and the subsequent activation of a TBK1‐AKT/mTOR signaling cascade.

### 3.7 ENPP2 Drives PD‐L1 Transcription by Promoting IRF3 Nuclear Translocation and Promoter Recruitment

To further delineate the molecular mechanism through which ENPP2 regulates PD‐L1 expression, we first sought to determine whether ENPP2‐mediated PD‐L1 upregulation occurs at the transcriptional level. Quantitative real‐time PCR (qRT‐PCR) analysis confirmed that genetic depletion of ENPP2 significantly reduced the mRNA expression of CD274 (the gene encoding PD‐L1) in both 786‐O and ACHN cells (Figure S6D), whereas ENPP2 overexpression robustly upregulated its transcription (Figure S6E). To further delineate the upstream signaling cascade driving this transcriptional activation, we investigated the potential physical and functional interaction between AKT and TBK1 within renal cancer cells. Immunofluorescence staining in 786‐O and ACHN cells revealed a strong cytoplasmic colocalization of AKT and TBK1 signals, as visualized by ImageJ Colocalization Finder analysis (Figure 7A,B). Reciprocal co‐immunoprecipitation (Co‐IP) in 293T cells co‐transfected with HA‐AKT and Flag‐TBK1 demonstrated a robust interaction (Figure 7C), which was consistently verified by endogenous Co‐IP in 786‐O and ACHN cells (Figure 7D,E). These findings establish that AKT physically associates with TBK1 in RCC cells, suggesting a direct signaling link connecting ENPP2–TBK1 activation to AKT/mTOR pathway engagement.

**FIGURE 7:**
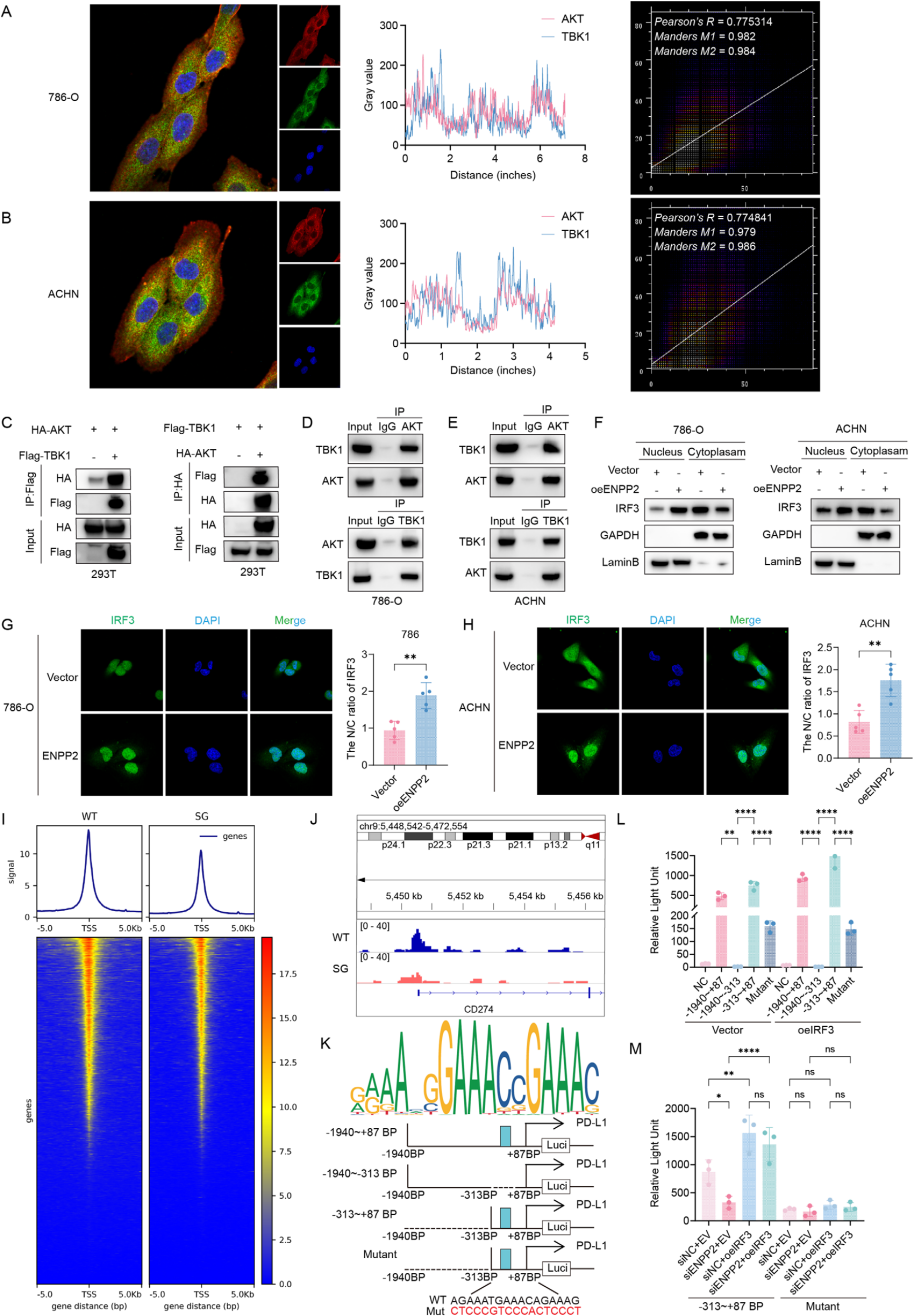
ENPP2 drives PD‐L1 transcription by promoting IRF3 nuclear translocation and promoter recruitment. (A, B) Representative immunofluorescence images and colocalization analysis of AKT (red) and TBK1 (green) in 786‐O (A) and ACHN (B) cells, with ImageJ Colocalization Finder analysis quantifying the degree of overlap (Pearson's R and Manders’ M1 and M2 values). (C) Co‐immunoprecipitation (Co‐IP) assays in 293T cells co‐transfected with HA‐AKT and Flag‐TBK1, showing a robust physical interaction between AKT and TBK1. (D, E) Endogenous Co‐IP assays in 786‐O (D) and ACHN (E) cells confirming the physiological physical interaction between AKT and TBK1. (F) Western blot analysis of nuclear (N) and cytoplasmic (C) fractions showing increased nuclear IRF3 accumulation in ENPP2‐overexpressing 786‐O and ACHN cells compared with controls. LaminB and GAPDH were used as nuclear and cytoplasmic markers, respectively. (G, H) Representative immunofluorescence images and quantification of the nuclear‐to‐cytoplasmic (N/C) ratio of IRF3 (green) in 786‐O (G) and ACHN (H) cells. (I) Heatmaps and average profile plots of IRF3 CUT&Tag sequencing data, illustrating global IRF3 binding density across transcription start sites (TSS) in wild‐type (WT) and ENPP2‐knockout (SG) 786‐O cells. (J) Integrative Genomics Viewer (IGV) tracks visualizing IRF3 CUT&Tag signals specifically at the CD274 promoter region in WT and SG cells. (K) Schematic representation of the JASPAR‐predicted IRF3 binding motif and the generated CD274 promoter luciferase reporter constructs, including full‐length, truncated, and motif‐mutated versions. (L) Relative luciferase activity of the indicated CD274 promoter constructs in cells co‐transfected with an empty vector or an IRF3 overexpression plasmid (oeIRF3). (M) Relative luciferase activity of the wild‐type (‐313 to +87 bp) and mutant CD274 promoter constructs in cells subjected to ENPP2 knockdown (siNC/siENPP2), IRF3 overexpression (EV/oeIRF3), or their combination. Quantitative in vitro data are presented as mean ± SD from *n* = 3 independent experiments. Statistical significance was determined using an unpaired two‐tailed Student's t‐test (G, H), or two‐way ANOVA with Tukey's multiple comparisons test (L, M). ns = not significant, *p < 0.05, **p < 0.01, ***p < 0.001, ****p < 0.0001.

Next, we investigated whether ENPP2 modulates the nuclear translocation of IRF3, a key transcription factor downstream of TBK1. Nuclear–cytoplasmic fractionation followed by Western blotting revealed a marked increase in nuclear IRF3 accumulation upon ENPP2 overexpression compared with controls (Figure 7F). Consistently, immunofluorescence staining confirmed enhanced nuclear localization of IRF3 in ENPP2‐overexpressing cells (Figure 7G,H), indicating that ENPP2 facilitates IRF3 phosphorylation and nuclear import.

To determine whether IRF3 directly binds to the CD274 promoter and mediates its transcriptional regulation, we used the JASPAR database to predict potential IRF3‐binding motifs within the CD274 regulatory region. The analysis identified a high‐confidence IRF3 consensus sequence (AGAAATGAAACAGAAAG) located approximately 1 kb upstream of the CD274 transcription start site, with a score of 17.187 and a relative score of 0.9009 (threshold 90%). To experimentally validate this prediction, we performed CUT&Tag sequencing and the results demonstrated that ENPP2 knockout markedly reduced IRF3 occupancy across promoter regions genome‐wide (Figure 7I). Visualization of CUT&Tag tracks in the Integrative Genomics Viewer (IGV) revealed a pronounced loss of IRF3 binding at the CD274 promoter region in ENPP2‐deficient cells (Figure 7J).

We subsequently utilized luciferase reporter assays to map the functional transcriptional regulation. Various CD274 promoter constructs were generated, including full‐length (‐1940 to +87 BP), truncated versions (‐1940 to ‐313 BP, ‐313 to +87 BP), and a mutant version with a disrupted IRF3‐binding motif (AGAAATGAAACAGAAAG to CTCCCGTCCCACTCCCT) (Figure 7K). When co‐transfected with an IRF3 overexpression plasmid (oeIRF3) in 293T cells, luciferase activity was significantly enhanced in the full‐length and ‐313 to +87 bp constructs, whereas no induction was observed in the mutant construct (Figure 7L). To validate the ENPP2‐dependent regulation, we used the ‐313 to +87 bp construct in combination with siENPP2 and/or IRF3 overexpression. Luciferase activity was significantly reduced following ENPP2 knockdown, and this effect was reversed upon IRF3 overexpression. No changes were observed in the mutant construct, confirming that ENPP2 regulates CD274 transcription through this specific IRF3 binding motif (Figure 7M). Importantly, we validated this epistatic relationship at the endogenous protein level. Immunoblot analysis revealed that the downregulation of PD‐L1 induced by ENPP2 depletion was robustly rescued by ectopic expression of a constitutively active IRF3 mutant (IRF3‐5D) in both 786‐O and ACHN cells (Figure S6F). Together, these data firmly establish IRF3 as the direct causal mediator of ENPP2‐driven PD‐L1 upregulation.

### 3.8 In Vivo Experiments Demonstrate That Targeting ENPP2 Improves T Cell Function and Enhances the Efficacy of Targeted‐Immune Combination Therapy

To evaluate the in vivo translational potential of targeting ENPP2 alongside cabozantinib, we first established an orthotopic syngeneic RCC model using ENPP2‐depleted (shENPP2) cells. Longitudinal bioluminescence imaging demonstrated that the combination strategy (shENPP2 + cabozantinib) exerted profoundly superior tumor‐suppressive effects compared to either monotherapy or the vehicle control (Figure S7A,B). Subsequent flow cytometric profiling of these orthotopic tumors revealed that while global intratumoral frequencies of CD4^+^ and CD8^+^ T cells remained stable across treatment groups (Figure S7C,D), ENPP2 knockdown specifically downregulated PD‐L1 expression on tumor cells in vivo (Figure S7E,F).

Building upon this genetic proof‐of‐concept, we next investigated the therapeutic potential of pharmacological ENPP2 inhibition in improving the efficacy of targeted–immune combination therapy. We established a renal cancer patient‐derived xenograft (PDX) model through serial passaging in NCG immunodeficient mice. For these in vivo pharmacological studies, the GLPG1690 was prioritized over the in vitro tool compound HA130 due to its superior systemic pharmacokinetic profile and advanced clinical translational potential. To reconstruct a functional immune microenvironment resembling the human tumor context, T cells were isolated from healthy donor peripheral blood, activated with dendritic cells (DCs) in vitro, and intravenously reinfused into tumor‐bearing mice (Figure S7G). The experimental timeline of immune reconstitution and subsequent drug administration is shown in Figure S7H. The ENPP2 enzymatic inhibitor GLPG1690 was used to pharmacologically suppress ENPP2 activity. T‐cell reinfusion combined with anti–PD‐1 antibody represented the immune therapy arm, while cabozantinib served as the TKI‐based targeted therapy. Tumor growth kinetics and endpoint tumor weights demonstrated that triple combination therapy (TKI + immune checkpoint blockade + ENPP2 inhibition) achieved the most pronounced tumor suppression, outperforming all monotherapy groups and the standard targeted–immune combination (Figure 8A–C). Flow cytometric profiling of dissociated tumor tissues revealed markedly reduced PD‐L1 expression on tumor cells in all ENPP2‐inhibited groups, consistent with our orthotopic and in vitro observations (Figure 8D,E). Notably, neither immune therapy nor TKI monotherapy altered PD‐L1 expression. Serum cytokine analysis showed significantly elevated IFN‐γ and TNF‐α levels in ENPP2‐targeted groups compared with controls (Figure 8F). Functional characterization of tumor‐infiltrating T cells across the four immune‐reconstituted treatment arms revealed a substantial increase in effector T‐cell markers (GZMB, Perforin, IFN‐γ, and TNF‐α) following ENPP2 inhibition (Figure 8G,H, Figure S7I–L). Conversely, exhaustion‐associated inhibitory receptors, including PD‐1, TIM‐3, and LAG‐3, were markedly downregulated (Figure 8I,J, Figure S7M). KI67 immunohistochemistry of tumor sections corroborated these results: ENPP2 inhibition alone reduced proliferative activity, whereas the ENPP2‐targeted triple combination therapy exhibited the lowest KI67 positivity, confirming a synergistic antitumor effect (Figure 8K). Taken together, these orthogonal in vivo findings demonstrate that targeting the ENPP2 axis reprograms the immunosuppressive tumor microenvironment, enhances T‐cell effector responses, and synergizes with targeted–immune combination therapy to overcome therapeutic resistance in renal cell carcinoma.

**FIGURE 8:**
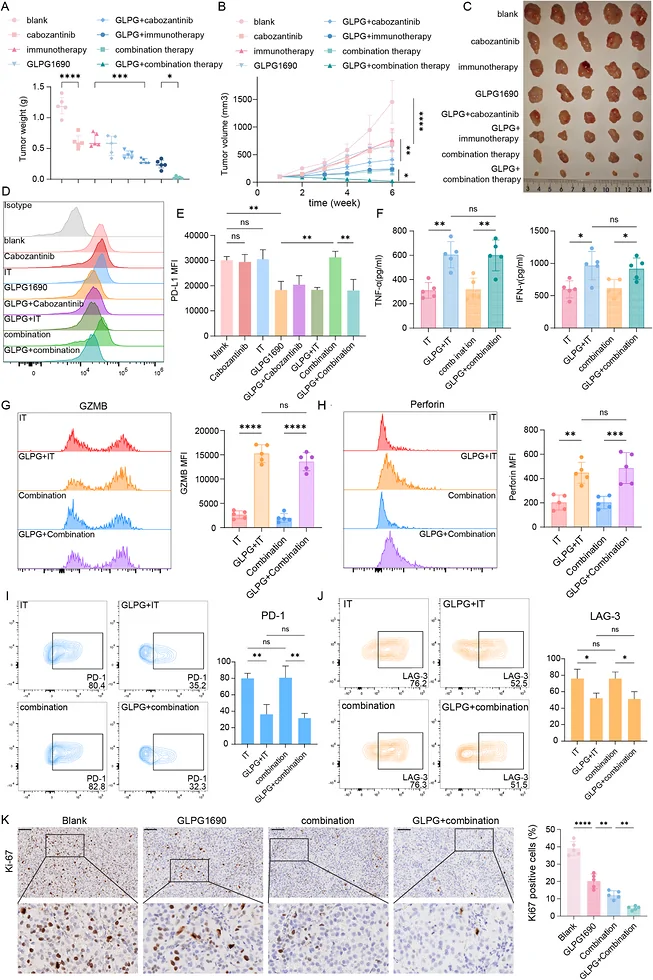
In vivo experiments demonstrate that targeting ENPP2 improves T cell function and enhances the efficacy of targeted‐immune combination therapy. (A–C) PDX subcutaneous tumor weights at the experimental endpoint (A), tumor volume changes recorded during the experiment (B), and representative tumor images (C) following treatment as outlined in the schematic. Immunotherapy refers to reinfusion of in vitro pre‐activated T cells and administration of αPD‐1 antibody. Combination therapy mimics clinical first‐line therapy, combining TKI with immune checkpoint inhibitor (ICI). (D, E) Representative flow cytometry histograms (D) and quantification of median fluorescence intensity (MFI) (E) for surface PD‐L1 expression on tumor cells dissociated from the excised xenografts. (F) Serum concentrations of TNF‐α and IFN‐γ in the indicated immune‐reconstituted murine cohorts, measured by ELISA. (G, H) Flow cytometric analysis and MFI quantification of the cytotoxic effector molecules Granzyme B (GZMB) (G) and Perforin (H) in tumor‐infiltrating CD8^+^ T cells. (I, J) Representative contour plots and quantitative analysis of the exhaustion markers PD‐1 (I) and LAG‐3 (J) on tumor‐infiltrating T cells. (K) Representative immunohistochemistry (IHC) images and quantification of the proliferation marker Ki‐67 in tumor sections from the specified treatment groups. Scale bars: 50 µm. All in vivo data are presented as mean ± SD with *n* = 5 independent biological replicates per group. Statistical significance was determined using one‐way ANOVA (A, E, F, G, H, I, J, K), or two‐way ANOVA (B), followed by Tukey's post hoc test. ns = not significant, *p < 0.05, **p < 0.01, ***p < 0.001, ****p < 0.0001.

## 4 Discussion

The therapeutic landscape for advanced renal cell carcinoma (RCC) has been redefined by the NCCN‐endorsed first‐line combination of tyrosine kinase inhibitors (TKIs) and immune checkpoint inhibitors [5]. Despite conferring significant survival benefits, RCC exhibits a paradoxically immunogenic yet highly immunosuppressive microenvironment; here, infiltrating T cells are frequently driven into functional exhaustion rather than exerting anti‐tumor immunity [8, 9]. Concurrently, while next‐generation TKIs such as cabozantinib have demonstrated superior clinical efficacy over sunitinib, therapeutic durability is persistently thwarted by intrinsic and acquired resistance [44, 45, 46]. Therefore, elucidating the molecular mechanisms that simultaneously drive the immunosuppressive tumor microenvironment and TKI resistance remains an urgent clinical challenge.

Historically, TKI resistance has been primarily attributed to compensatory kinase activation or secondary genomic alterations. Our findings expand this paradigm by highlighting a fundamental metabolic rewiring. Rather than a uniform global increase in lipids, our targeted metabolomics demonstrated a specific intracellular accumulation of the highly bioactive unsaturated species LPA (18:1) in cabozantinib‐resistant cells. This metabolic shift provides a robust biochemical basis for sustained oncogenic signaling. Intriguingly, our single‐cell RNA sequencing analysis revealed that the LPA receptor, LPAR1, is highly enriched in the CD44^+^CD24^−^ malignant subpopulation, a subset widely recognized for its cancer stem cell (CSC)‐like characteristics [38, 39]. Coupled with the observation from CancerSea datasets depicting a metabolic tug‐of‐war, where ENPP2 promotes hypoxia and stemness while the LPA‐catabolizing enzyme PLPP1 exerts the opposite effect. Our data provide a highly plausible mechanistic explanation for how the ENPP2–LPA axis sustains a resilient, dedifferentiated tumor state capable of evading targeted therapies.

Crucially, this metabolic‐driven resistance is executed through profound intracellular signaling rewiring. While the AKT/mTOR pathway is frequently hyperactivated in clear cell RCC (ccRCC), therapies targeting this node often yield suboptimal clinical efficacy [47, 48]. It is noteworthy that the 786‐O cell line utilized in our study harbors a known PTEN mutation, conferring a constitutively high basal level of AKT/mTOR activation. Strikingly, targeting the ENPP2‐LPAR1 axis effectively abrogated this signaling cascade despite the PTEN‐deficient background. This establishes ENPP2 not merely as a bystander, but as an indispensable upstream rheostat for AKT/mTOR hyperactivation in ccRCC, highlighting the therapeutic potential of combining ENPP2 inhibitors with mTOR inhibitors in RCC treatment to bypass intrinsic genetic resistance, further investigation into the combination of ENPP2 inhibitors with mTOR inhibitors is warranted to evaluate their therapeutic potential in RCC.

Beyond tumor‐intrinsic survival, our mechanistic investigations uncover a novel signaling cascade linking lipid metabolism to immune checkpoint regulation. Activation of the cGAS‐STING pathway leads to production of type I interferons and the expression of immune checkpoint molecules like PD‐L1, facilitating immune evasion [49, 50, 51]. While previous studies have illustrated the dual, context‐dependent functions of the cGAS‐STING/TBK1‐IRF3 pathway in either promoting immune surveillance or facilitating immune evasion via PD‐L1 induction [52, 53], we define the specific metabolic trigger for this cascade in RCC. By demonstrating that the AKT‐TBK1 complex acts as a critical signal transducer, we mapped a continuous axis from extracellular LPA to transcriptional reprogramming. Importantly, the integration of our mutant promoter assays with endogenous epistatic rescue experiments firmly establishes IRF3 as the obligate causal mediator bridging the ENPP2–LPA axis to PD‐L1 upregulation. This delineates an intricate signaling network where TBK1 functions as a nodal kinase, simultaneously driving therapeutic resistance via AKT/mTOR and immune evasion via IRF3‐dependent PD‐L1 transcription.

Within the broader tumor microenvironment, our data position ENPP2 as a dual‐action metabolic checkpoint. Utilizing the TcellSI algorithm [43], we uncovered a profound shift toward CD8^+^ T‐cell exhaustion in ENPP2‐high tumors. Mechanistically, our co‐culture rescue assays reveal that this immune suppression is bipartite. While pharmacologic or genetic blockade of the PD‐1/PD‐L1 axis significantly diminished the survival advantage of ENPP2‐overexpressing cells, a residual suppression of T‐cell cytotoxicity persisted. This observation, coupled with the strong correlation between LPAR5 and exhaustion markers (PD‐1, LAG‐3, CTLA‐4), strongly indicates that while ENPP2‐driven PD‐L1 induction contributes substantially to immune evasion, the secreted metabolite LPA also exerts direct inhibitory effects on T cells. This perfectly aligns with prior reports of LPAR5‐mediated T‐cell suppression [27, 28, 54], illustrating a comprehensive strategy by which RCC tumors disarm cytotoxic immunity.

Despite the robust mechanistic insights provided by our study, certain limitations remain. First, our in vivo findings heavily rely on syngeneic and humanized murine models. Future investigations employing large‐scale, prospective clinical cohorts with paired pre‐ and post‐treatment ccRCC biopsies are essential to clinically validate the dynamic regulatory role of the ENPP2‐PD‐L1 axis. Second, given the pleiotropic nature of LPA signaling, the specific impact of ENPP2 blockade on other critical components of the tumor microenvironment, such as myeloid‐derived suppressor cells, remains to be fully elucidated. Lastly, while the preclinical synergy of targeting ENPP2 alongside cabozantinib and anti‐PD‐1 is compelling, rigorously designed clinical trials are required to confirm the safety and definitive therapeutic efficacy of this combination strategy in patients.

In conclusion, our study uncovers an intricate interplay wherein the ENPP2–LPA axis leverages the AKT/mTOR and TBK1/IRF3 signaling networks to orchestrate TKI resistance and dual‐mode immune evasion in RCC (Figure 9). These findings provide a compelling biological and translational rationale for combining LPA‐pathway inhibition (e.g., GLPG1690) with TKIs and PD‐1/PD‐L1 blockade, offering a promising multifaceted strategy to fully reinvigorate antitumor immunity and overcome therapeutic resistance in advanced RCC.

**FIGURE 9:**
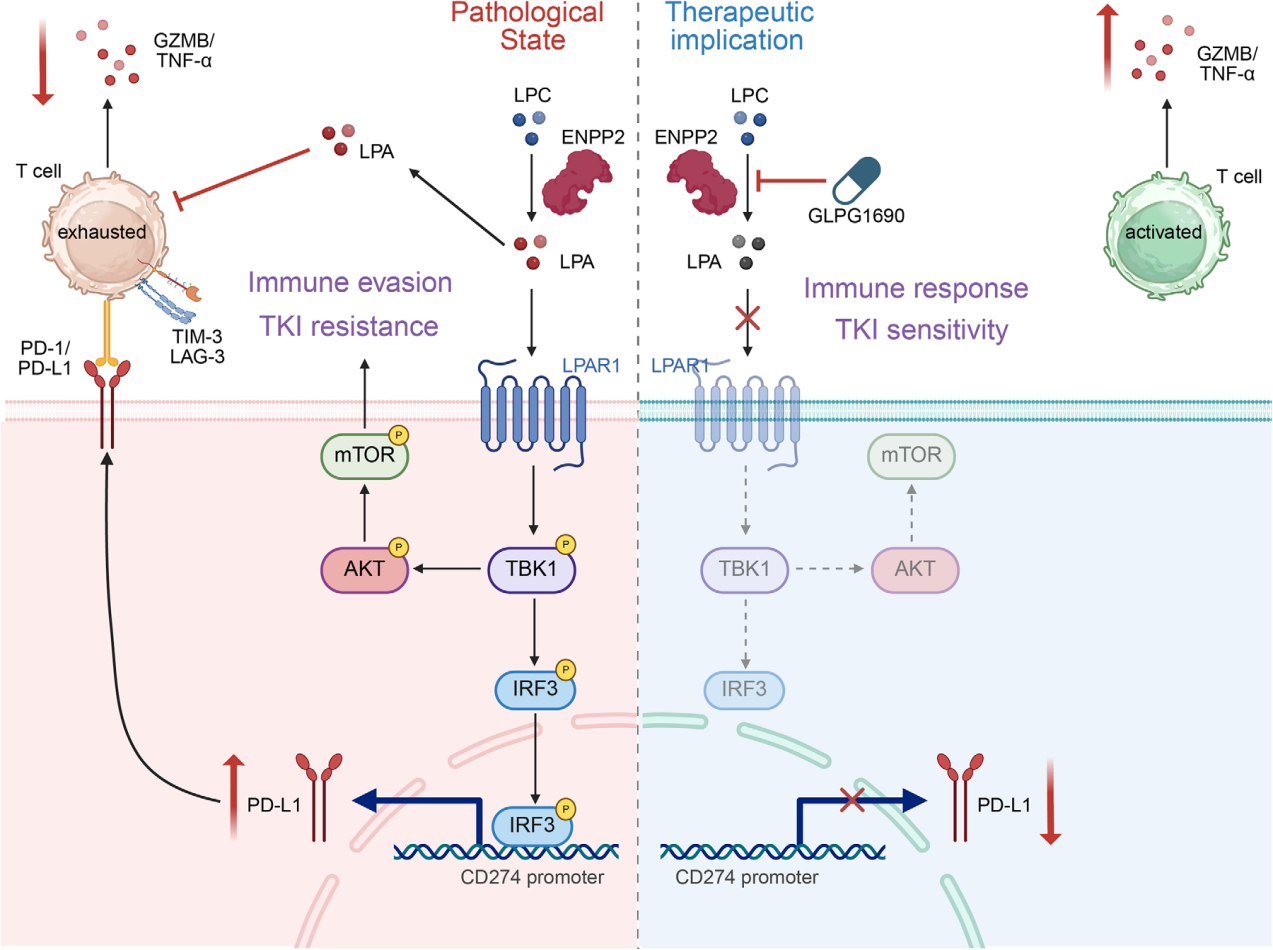
Schematic Illustration of the ENPP2–LPA Axis Driving TKI Resistance and Immune Evasion in Renal Cell Carcinoma. ENPP2 (Autotaxin) catalyzes the conversion of LPC to LPA. In the tumor microenvironment, LPA engages LPAR1 on renal cancer cells to activate TBK1. This signaling bifurcates to: (1) stimulate the AKT–mTOR cascade, conferring resistance to tyrosine kinase inhibitors (TKIs); and (2) induce IRF3 phosphorylation and nuclear translocation. Nuclear IRF3 transactivates the CD274 promoter, upregulating surface PD‐L1. Subsequent PD‐L1/PD‐1 engagement, further exacerbated by the accumulation of immunosuppressive LPA, triggers T‐cell exhaustion (characterized by increased TIM‐3/LAG‐3 and impaired GZMB/TNF‐α secretion). Conversely, pharmacological blockade of ENPP2 with GLPG1690 abrogates LPA synthesis, silencing downstream LPAR1–TBK1 signaling and alleviating extracellular suppressive stress on T cells. This intervention downregulates PD‐L1, restores TKI sensitivity, and reinvigorates cytotoxic T‐cell effector functions.

## Author Contributions

Conceptualization: **Jinchen Luo**, **Junjie Cen**, **Yanping Liang**. Data Curation: Jinchen Luo, **Zheyu Ai**. Formal Analysis: **Yinghan Wang**, **Yong Huang**. Funding Acquisition: Junjie Cen, Yanping Liang, **Wei Chen**, **Junhang Luo**, **Lei Tan**, **Xi Liu**, Yong Huang. Investigation: Jinchen Luo, **Hansen Lin**, Yanping Liang. Methodology: Jinchen Luo, Hansen Lin, **Jiajie Chen**. Project administration: Junjie Cen, Junhang Luo. Resources: Wei Chen, Junhang Luo, Yanping Liang. Software: **Mingjie Lin**, **Minyu Chen**, **Wuyuan Liao**. Supervision: Wei Chen, Junhang Luo, Yanping Liang. Validation: Hansen Lin, Lei Tan, Xi Liu. Visualization: **Haoqian Feng**, **Xinwei Zhou**. Writing – original Draft: Jinchen Luo, Hansen Lin, Haoqian Feng, Lei Tan, Xi Liu. Writing – Review & Editing: Jinchen Luo, Wei Chen, Junhang Luo, Yanping Liang.

## Funding

This work was supported by the Natural Science Foundation of China (No. 82302613, Yanping Liang; No. 82203437, Junjie Cen; No. 82303388, Lei Tan; No. 82272862, Wei Chen; No. 82473417, Wei Chen; No. 82373433, Junhang Luo); Guangzhou Basic Research Program‐City‐University (Hospital)‐Enterprise Joint Funding Project (No. 2025A03J3619, Xi Liu) and Guangxi Natural Science Foundation Program (No. 2025GXNSFAA069290, Yong Huang)

## Ethics Approval and Consent to Participate

The collection and use of all patient samples and clinical data were approved by the Institutional Ethics Committee for Clinical Research of the First Affiliated Hospital of Sun Yat‐sen University (Guangzhou, China). Informed consent was obtained from the patients.

## Conflicts of Interest

The authors declare no conflicts of interest.

## Supporting information




**Supporting File**: advs76352‐sup‐0001‐SuppMat.docx.

## Data Availability

The datasets used and/or analyzed during the current study are available from the corresponding author on reasonable request.
