# Supplementary material for: Targeting the ATX‐LPA Axis Overcomes TKI Resistance and Immunosuppression in Renal Cell Carcinoma via Dual Inhibition of AKT/mTOR and TBK1/IRF3 Pathways
Source: Adv Sci (Weinh). 2026 Jun 29:e76352. Online ahead of print. doi: 10.1002/advs.76352 (PMC13336963; doi:10.1002/advs.76352)
Supplement: Supplementary file 1 — Supporting File: advs76352‐sup‐0001‐SuppMat.docx. [file ADVS-9999-e76352-s001.docx]

Supplementary Information for

**Targeting the ATX-LPA Axis Overcomes TKI Resistance and Immunosuppression in Renal Cell Carcinoma via Dual Inhibition of AKT/mTOR and TBK1/IRF3 Pathways**

**Jinchen Luo**^a †^**,** **Hansen Lin**^a †^**, Haoqian Feng**^a †^**, Lei Tan^c^** ^†^**, Xi Liu^d,e^** ^†^**, Yong Huang^a,f^, Junjie Cen**^a^**, Jiajie Chen^g^, Xinwei Zhou**^a^**, Mingjie Lin**^a^**, Wuyuan Liao**^h^**, Zheyu Ai**^a^**, Minyu Chen**^a^**, Yinghan Wang**^a^**, Wei Chen** ^a *^**, Junhang Luo**^a *^**, Yanping Liang**^b *^

^a^ Department of Urology, The First Affiliated Hospital of Sun Yat-sen University, No. 58, Zhongshan Road II, Guangzhou, 510080, China

^b^ Department of Laboratory Medicine, The First Affiliated Hospital of Sun Yat-sen University, No. 58, Zhongshan Road II, Guangzhou, 510080, China

^c^ The Fourth Affiliated Hospital of Guangzhou Medical University, Urology Department, Guangzhou, China

^d^ Huadu District People’s Hospital of Guangzhou, Guangzhou, 510800, China

^e^ Huadu Institute of Medical Sciences, Guangzhou, 510000, China.

^f^ Guangxi Hospital Division of The First Affiliated Hospital, Sun Yat-sen University, Nanning, 530000, China

^g^ Department of Pediatrics, The First Affiliated Hospital of Sun Yat-sen University, No.58, Zhongshan Road II, Guangzhou, 510080, China

^h^ Department of Urology, Cancer Hospital Chinese Academy of Medical Sciences, Shenzhen Center, Shenzhen, China

† Jinchen Luo, Hansen Lin, Haoqian Feng, Lei Tan and Xi Liu contributed equally to this work.

* Correspondence should be addressed to Wei Chen ([chenw3@mail.sysu.edu.cn](mailto:chenw3@mail.sysu.edu.cn)), Junhang Luo ([luojunh@mail.sysu.edu.cn](mailto:luojunh@mail.sysu.edu.cn)) and Yanping Liang (liangyp9@mail.sysu.edu.cn)

**Table of Contents**

**Supplementary Figures:**

**Figure S1 – Figure S7**

**Supplementary Tables:**

**Table S1. Baseline information of the patient cohorts**

**Table S2. Targeted sequences of siRNA and shRNA**

**Table S3. Reagents and antibodies**

**Table S4. Databases and genesets**

**Table S5. Clinical and pathological characteristics of patient cohorts used for Single-cell RNA sequencing analysis.**

**
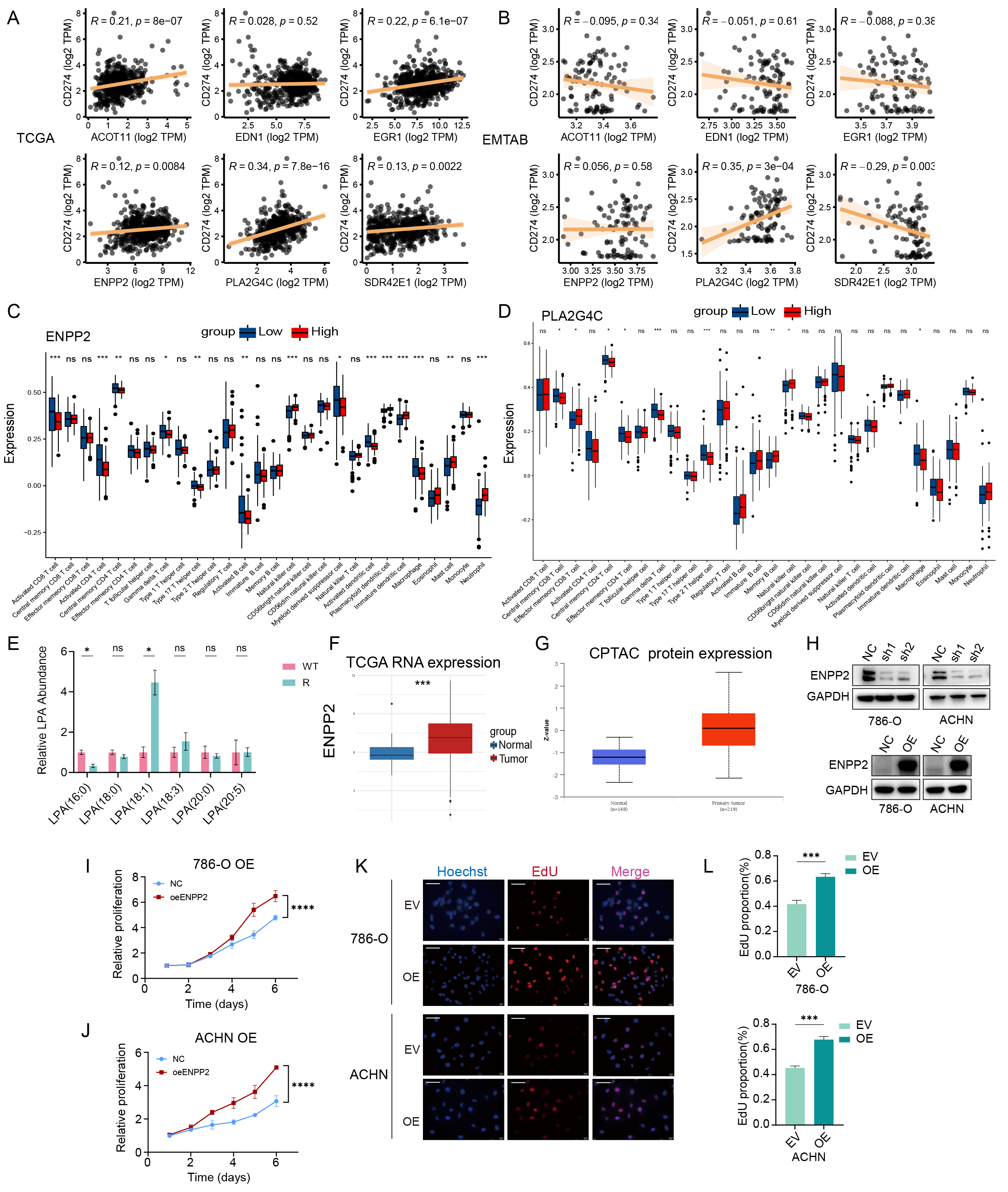
**

**Figure S1**

**(A, B)** Scatter plots demonstrating the correlation between CD274 (PD-L1) mRNA expression and six prognostic-related lipid metabolism genes (ACOT11, EDN1, EGR1, ENPP2, PLA2G4C, SDR42E1) in the TCGA-KIRC cohort **(A)** and the E-MTAB cohort **(B)**. Spearman correlation coefficients (*R*) and *P*-values are indicated.

**(C, D)** Single-sample Gene Set Enrichment Analysis (ssGSEA) comparing the infiltration abundances of various immune cell subsets between ENPP2-high and low expression groups **(C)**, and PLA2G4C-high and low expression groups **(D)** in the TCGA cohort.

**(E)** Relative abundance of specific lysophosphatidic acid (LPA) molecular species in wild-type (WT) and cabozantinib-resistant (R) RCC cells, quantified by targeted lipidomics.

**(F, G)** Basal ENPP2 mRNA expression in the TCGA cohort **(F)** and ENPP2 protein expression in the CPTAC cohort **(G)**, comparing normal tissues and primary RCC tumors.

**(H)** Immunoblot validation of ENPP2 stable knockdown (sh1, sh2) and overexpression (OE) efficiency in 786-O and ACHN cell lines, with GAPDH serving as the loading control.

**(I, J)** Cell proliferation curves of 786-O **(I)** and ACHN **(J)** cells following ENPP2 overexpression (oeENPP2) compared to the negative control (NC), evaluated by CCK-8 assays over a 6-day period.

**(K, L)** Representative fluorescence images **(K)** and quantification **(L)** of EdU incorporation assays demonstrating enhanced proliferation in ENPP2-overexpressing (OE) RCC cells compared to empty vector (EV) controls. Scale bars: 50 μm.

Quantitative *in vitro* data are presented as mean ± SD from n = 3 independent experiments. Statistical significance was determined using an unpaired two-tailed Student’s t-test **(C, D, E, F, G, L)**, or two-way ANOVA with Tukey's multiple comparisons test **(I, J)**. ns = not significant, **P* < 0.05, ***P* < 0.01, ****P* < 0.001, *****P* < 0.0001.

**
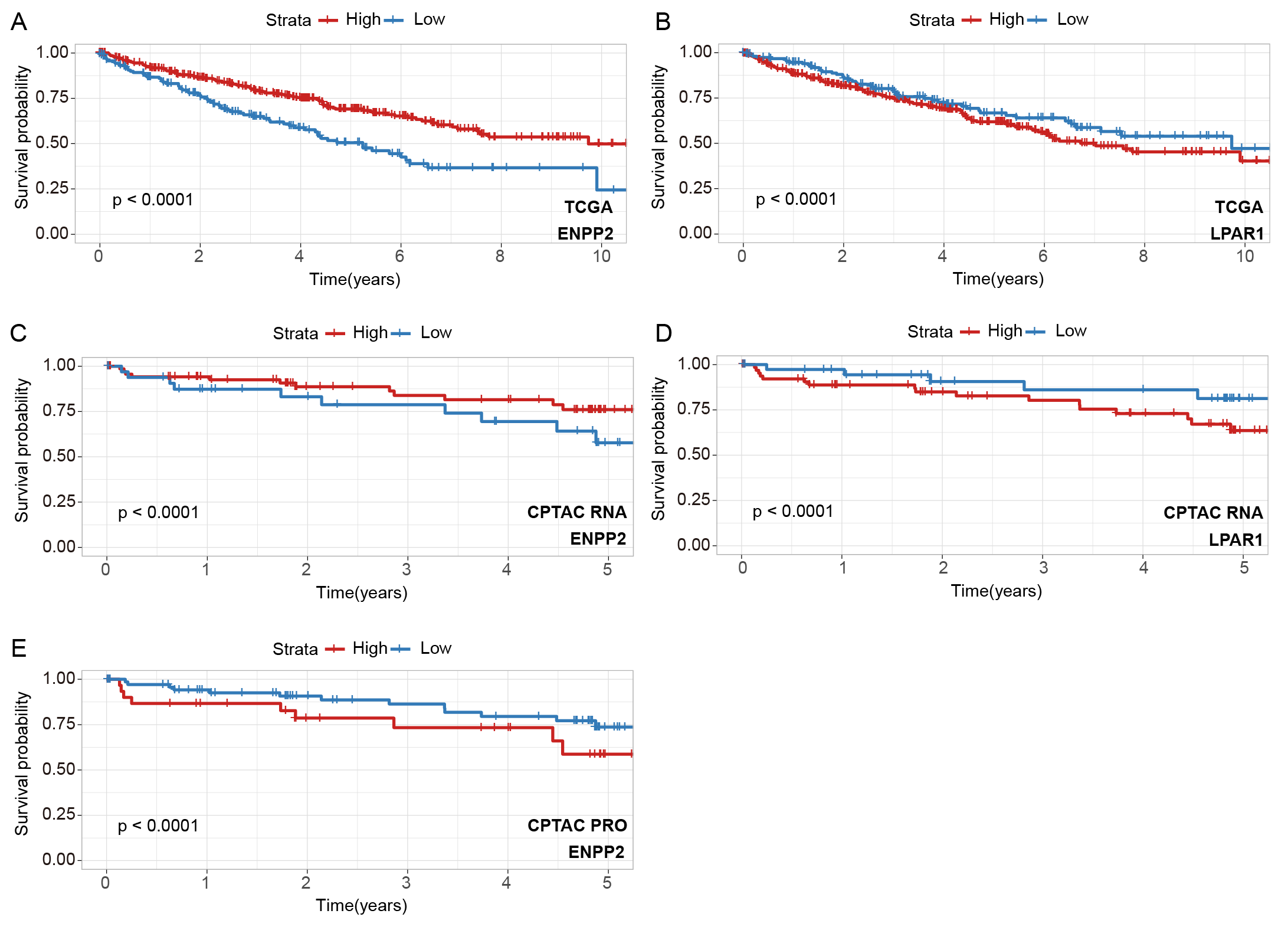
**

**Figure S2**

**(A, B)** Kaplan-Meier overall survival (OS) curves of patients in the TCGA-KIRC cohort, stratified by high versus low mRNA expression levels of ENPP2 **(A)** and LPAR1 **(B)**.

**(C–E)** Kaplan-Meier survival analyses of patients in the CPTAC cohort based on the expression levels of ENPP2 mRNA **(C)**, LPAR1 mRNA **(D)**, and ENPP2 protein **(E)**.

Statistical significance between survival curves was determined using the log-rank test.


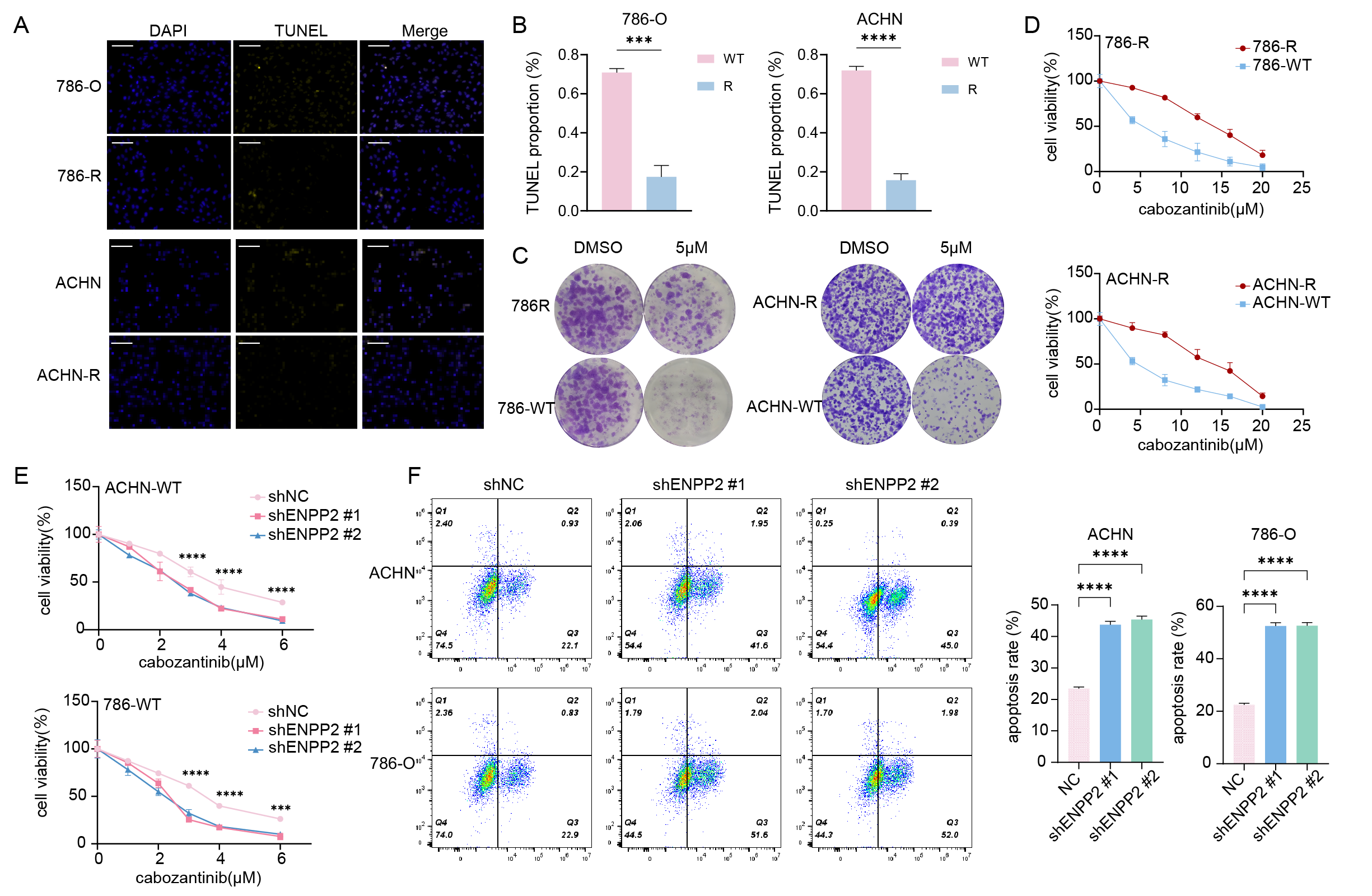


**Figure S3**

**(A, B)** Representative fluorescence images of TUNEL staining (A) and quantification (B) of apoptosis in wild-type (WT) and cabozantinib-resistant (R) 786-O and ACHN cells. Scale bars: 50 μm.

**(C)** Representative images of colony formation assays for WT and R cells treated with DMSO or 5 μM cabozantinib.

**(D)** Cell viability evaluated by CCK-8 assays in WT and R cells across a cabozantinib concentration gradient.

**(E)** CCK-8 cell viability assays of WT ACHN and 786-O cells following ENPP2 knockdown (shNC, shENPP2 #1, and #2) across a cabozantinib concentration gradient.

**(F)** Representative flow cytometry plots and quantitative analysis of apoptosis rates in WT RCC cells following ENPP2 depletion.

Data are presented as mean ± SD from n = 3 independent experiments. Statistical significance was determined using an unpaired two-tailed Student’s t-test **(B)**, two-way ANOVA **(D, E)**, or one-way ANOVA **(F)** with Tukey’s multiple comparisons test **(F)**. ****P* < 0.001, *****P* < 0.0001.


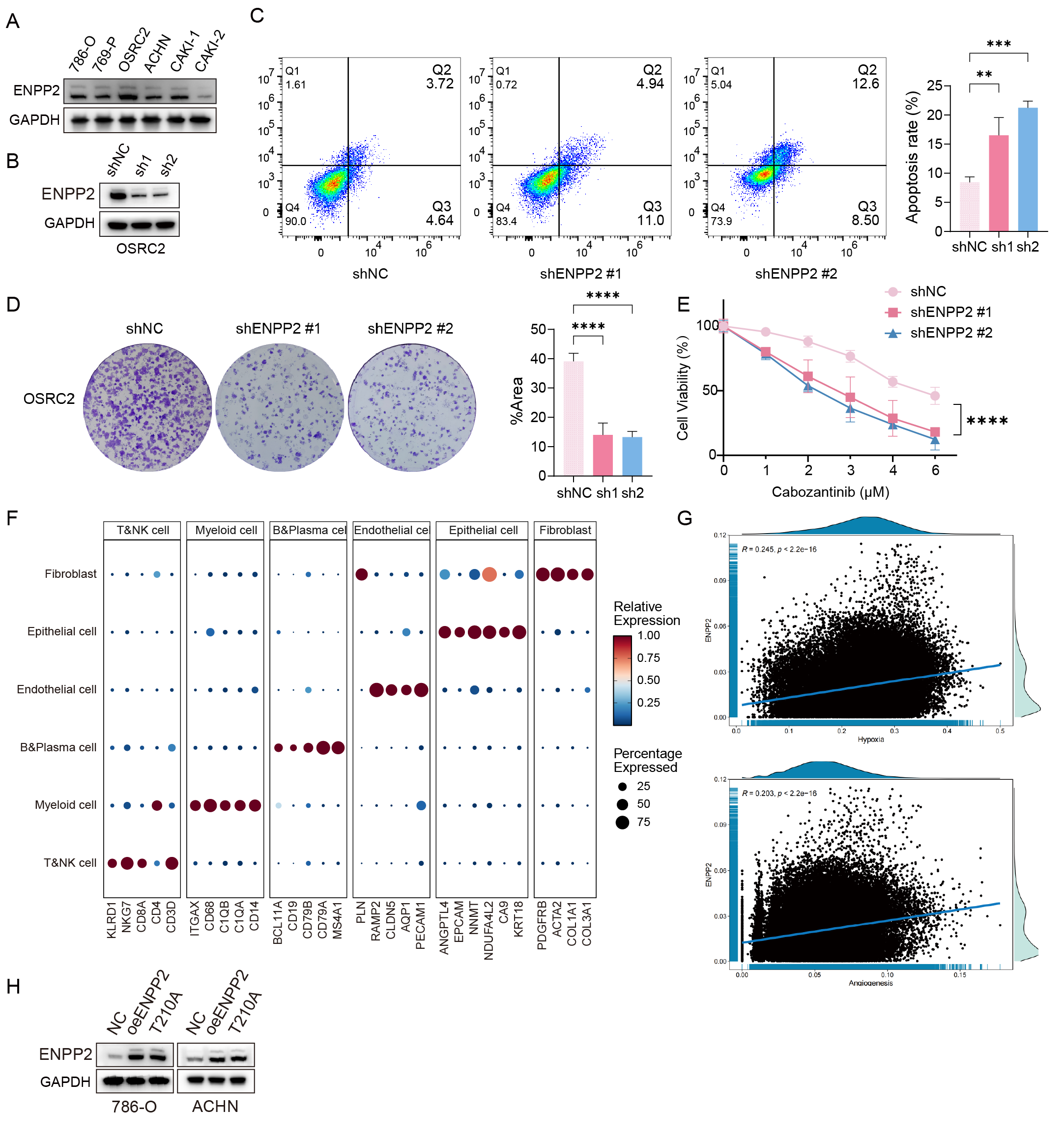


**Figure S4**

**(A)** Immunoblot analysis of basal ENPP2 protein expression across six human ccRCC cell lines (786-O, 769-P, OSRC2, ACHN, CAKI-1, CAKI-2).

**(B)** Immunoblot validation of ENPP2 knockdown efficiency (shNC, sh1, sh2) in the OSRC2 cell line.

**(C, D)** Representative flow cytometry plots **(C)** and colony formation images **(D)** evaluating the apoptosis rate and proliferation, respectively, in shNC and shENPP2 OSRC2 cells under cabozantinib treatment. Quantitative analyses are shown on the right.

**(E)** Cell viability evaluated by CCK-8 assays in OSRC2 cells subjected to ENPP2 knockdown across a cabozantinib concentration gradient.

**(F)** Dot plot illustrating the expression distribution of canonical marker genes across six major cell lineages (Fibroblast, Epithelial cell, Endothelial cell, B & Plasma cell, Myeloid cell, and T & NK cell) identified in the single-cell RNA sequencing (scRNA-seq) dataset.

**(G)** Scatter plots depicting the positive correlation between ENPP2 expression and Hypoxia (top) or Angiogenesis (bottom) signature scores.

**(H)** Immunoblot analysis confirming the stable ectopic expression of wild-type ENPP2 (oeENPP2) and the specific mutant construct (T210A) in 786-O and ACHN cell lines.

Quantitative *in vitro* data are presented as mean ± SD from n = 3 independent experiments. Statistical significance was determined using one-way ANOVA with Tukey’s multiple comparisons test **(C, D)** or two-way ANOVA **(E)** with Tukey’s multiple comparisons test. ***P* < 0.01, ****P* < 0.001, *****P* < 0.0001.

**
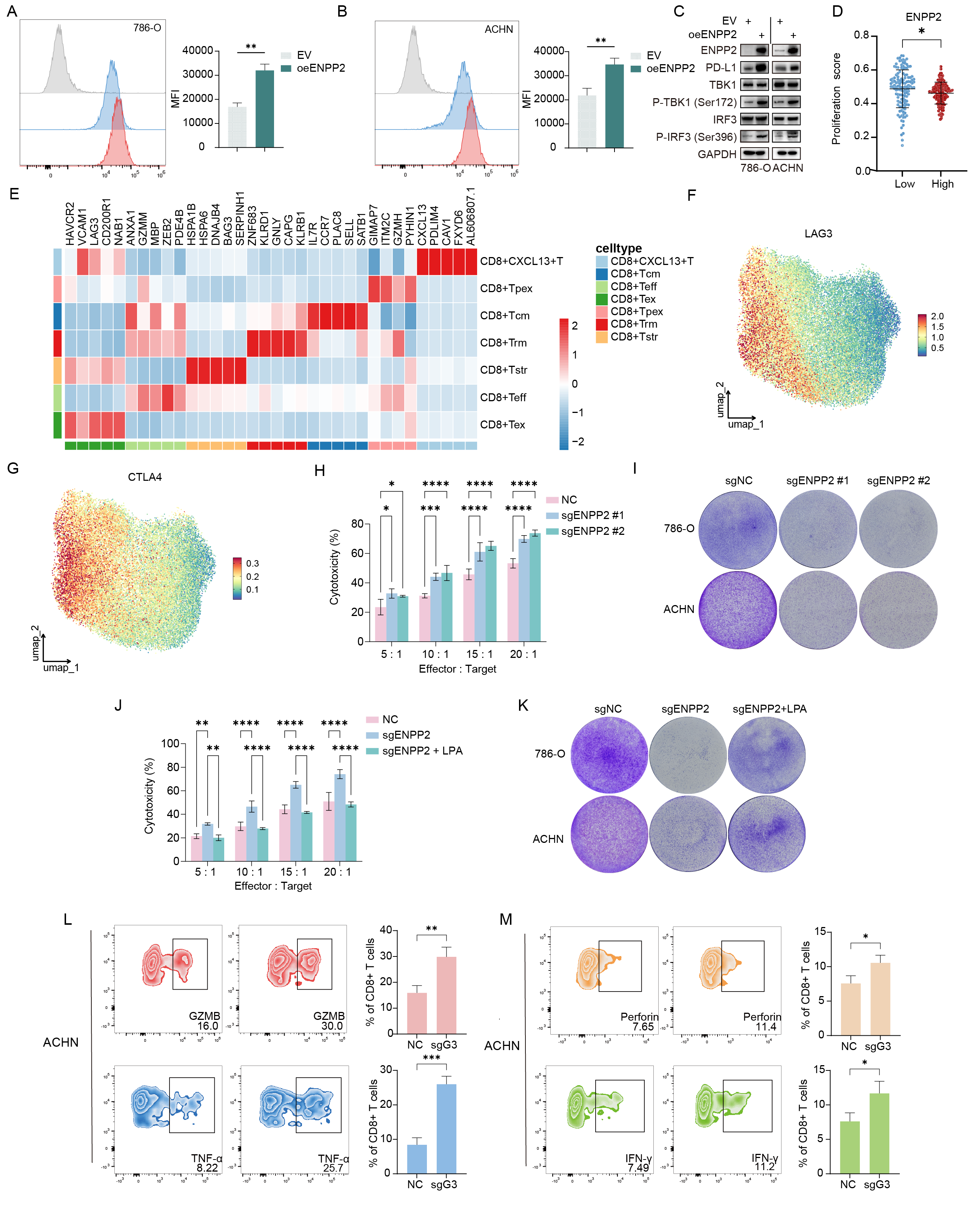
Figure S5**

**(A, B)** Flow cytometric analysis and quantification of median fluorescence intensity (MFI) for surface PD-L1 expression in 786-O **(A)** and ACHN **(B)** cells following ENPP2 overexpression (oeENPP2) compared to empty vector (EV) controls.

**(C)** Immunoblot analysis evaluating the expression of ENPP2, PD-L1, and the activation status of the TBK1/IRF3 signaling cascade (including P-TBK1 Ser172 and P-IRF3 Ser396) in EV and oeENPP2 RCC cells.

**(D)** Comparison of the proliferation score between ENPP2-low and ENPP2-high groups.

**(E)** Heatmap illustrating the distinct expression patterns of immune-related marker genes across diverse CD8⁺ T cell subpopulations identified in the scRNA-seq dataset.

**(F, G)** Uniform Manifold Approximation and Projection (UMAP) plots displaying the distribution of the exhaustion markers LAG3 **(F)** and CTLA4 **(G)**.

**(H, I)** T cell-mediated cytotoxicity evaluated by lactate dehydrogenase (LDH) release assays across varying effector-to-target (E:T) ratios **(H)**, and representative macroscopic images of surviving crystal violet-stained tumor cells from parallel co-culture colony formation assays **(I)**. CD8⁺ T cells were co-cultured with control (sgNC) or ENPP2-knockout (sgENPP2 #1 and #2) RCC cells.

**(J, K)** LDH release assays **(J)** and representative macroscopic images of surviving crystal violet-stained tumor cells **(K)** demonstrating that exogenous LPA supplementation functionally rescues the enhanced T cell-mediated killing susceptibility of ENPP2-knockout cells.

**(L, M)** Representative flow cytometry plots and quantitative analysis of effector molecule production, including GZMB and TNF-α **(L)**, as well as Perforin and IFN-γ **(M)**, in CD8⁺ T cells following co-culture with control (NC) or ENPP2-depleted (SG) ACHN cells.

Quantitative *in vitro* data are presented as mean ± SD from n = 3 independent experiments. Statistical significance was determined using an unpaired two-tailed Student’s t-test **(A, B, D, L, M)**, or two-way ANOVA **(H, J)** with Tukey’s multiple comparisons test. **P* < 0.05, ***P* < 0.01, ****P* < 0.001, *****P* < 0.0001.

**
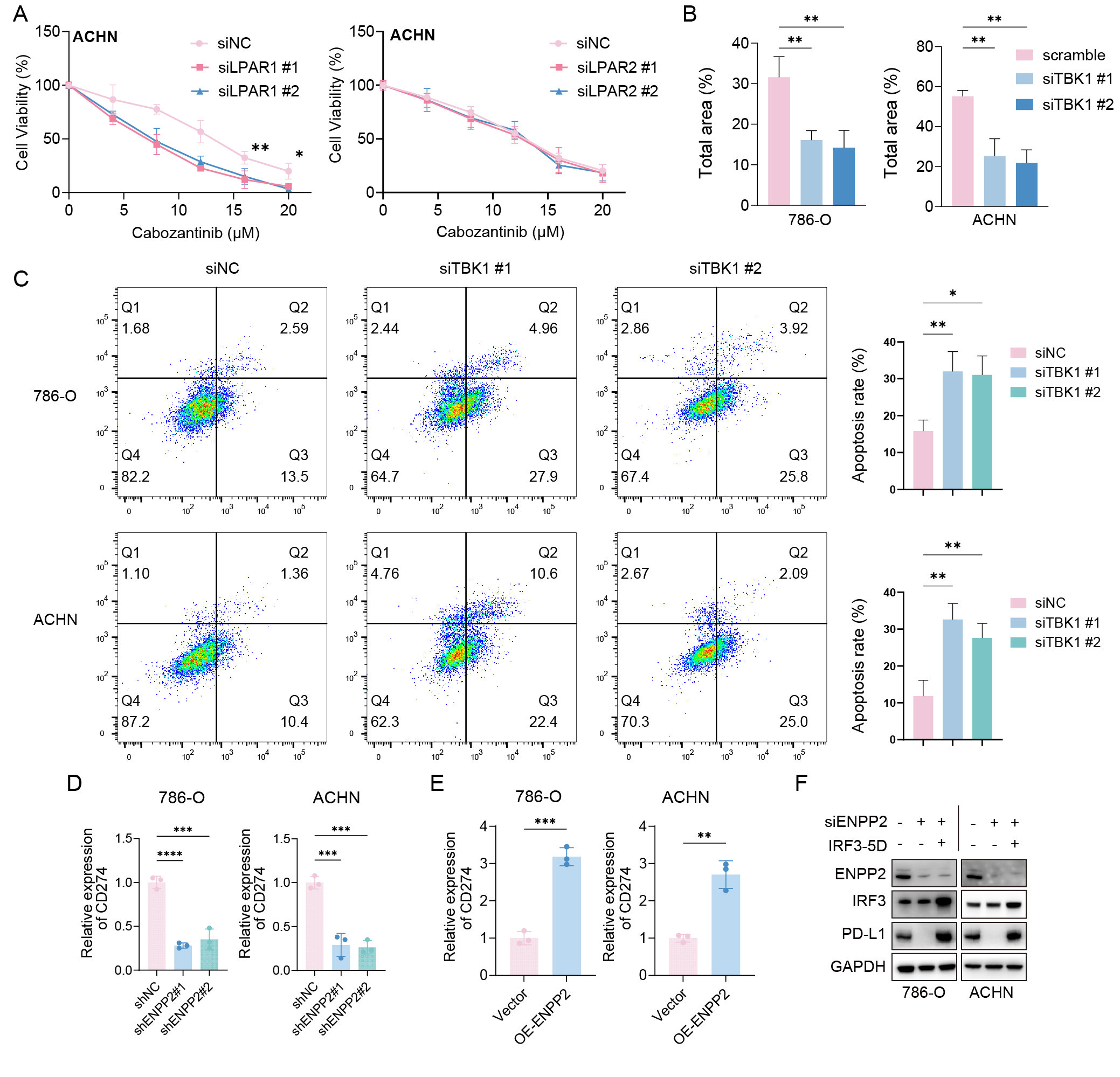
**

**Figure S6**

**(A)** Cell viability assessed by CCK-8 assays in ACHN cells following knockdown of LPAR1 or LPAR2 across a cabozantinib concentration gradient.

**(B)** Quantification of the total colony formation area in 786-O and ACHN cells following TBK1 knockdown.

**(C)** Representative flow cytometry plots and quantitative analysis of the apoptosis rate in 786-O and ACHN cells subjected to TBK1 depletion under cabozantinib treatment.

**(D, E)** Relative mRNA expression of CD274 (PD-L1) determined by RT-qPCR in 786-O and ACHN cells following ENPP2 knockdown **(D)** or ENPP2 overexpression (OE-ENPP2) **(E)**.

**(F)** Immunoblot analysis evaluating the expression of ENPP2, IRF3, and PD-L1 in 786-O and ACHN cells.

Quantitative *in vitro* data are presented as mean ± SD from n = 3 independent experiments. Statistical significance was determined using an unpaired two-tailed Student’s t-test **(E)**, one-way ANOVA **(B, C, D)**, or two-way ANOVA **(A)** with Tukey’s multiple comparisons test. **P* < 0.05, ***P* < 0.01, ****P* < 0.001, *****P* < 0.0001.

**
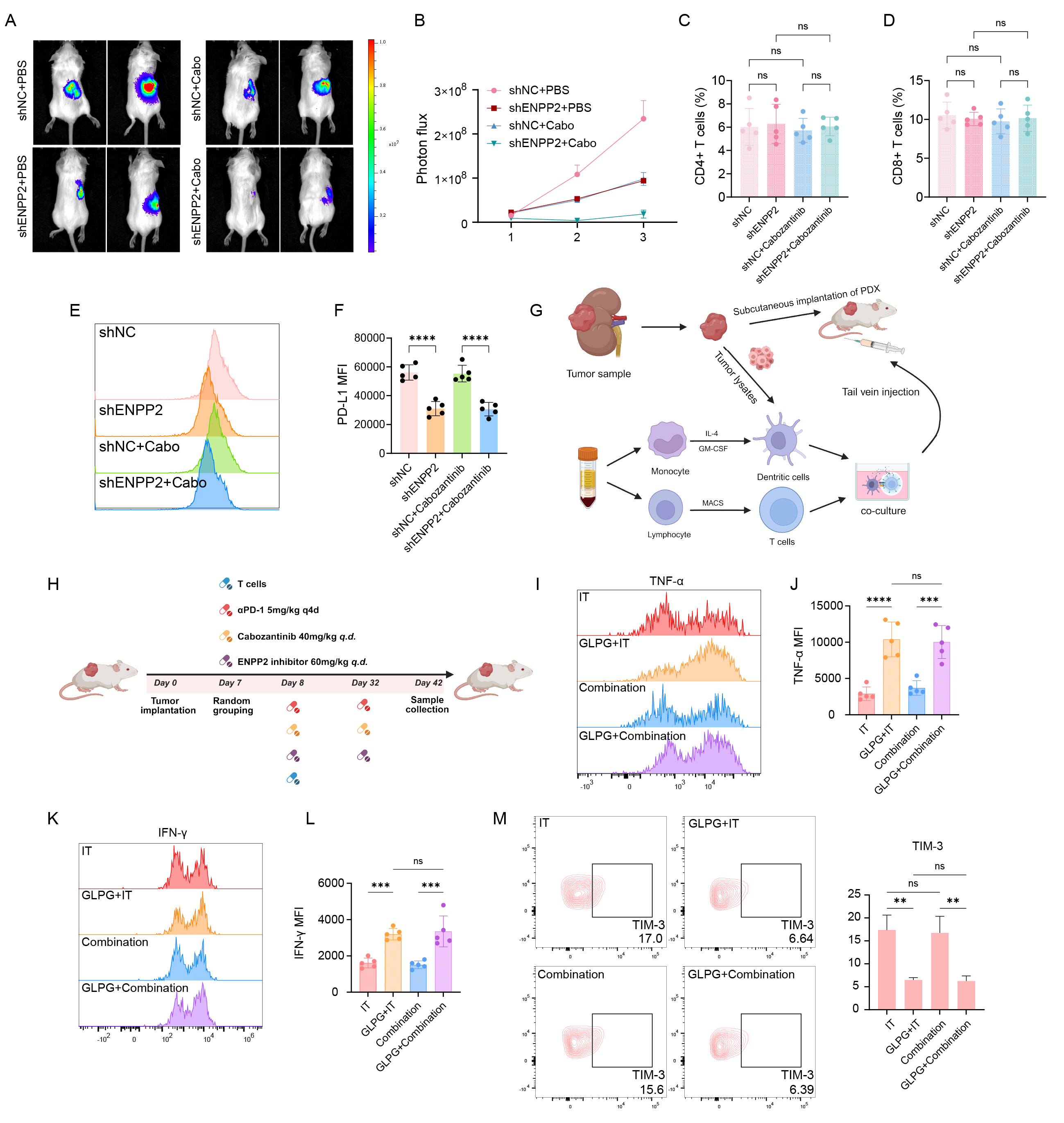
**

**Figure S7**

**(A, B)** Representative bioluminescence images **(A)** and quantitative tumor growth curves **(B)** of orthotopic RCC xenografts. Mice bearing shNC or ENPP2-knockdown (shENPP2) tumors were treated with vehicle (PBS) or cabozantinib (Cabo).

**(C, D)** Quantitative flow cytometry analysis of the intra-tumoral infiltration proportions of CD4⁺ **(C)** and CD8⁺ **(D)** T cells across the indicated treatment groups.

**(E, F)** Representative flow cytometry histograms **(E)** and quantitative MFI analysis **(F)** of surface PD-L1 expression on tumor cells isolated from the *in vivo* xenografts.

**(G)** Schematic illustration of the establishment of the patient-derived xenograft (PDX) model combined with autologous T cell co-transfer.

**(H)** Schematic diagram depicting the *in vivo* treatment timeline and regimens.

**(I–L)** Representative flow cytometry histograms and corresponding MFI quantification for the effector cytokines TNF-α **(I, J)** and IFN-γ **(K, L)** in tumor-infiltrating CD8⁺ T cells across the specified treatment groups (IT, GLPG+IT, Combination, and GLPG+Combination).

**(M)** Representative flow cytometry plots and quantitative analysis of the exhaustion marker TIM-3 on tumor-infiltrating CD8⁺ T cells.

Quantitative *in vivo* and flow cytometry data are presented as mean ± SD. n = 5 mice per group. Statistical significance was determined using one-way ANOVA **(C, D, F, J, L, M)** or two-way ANOVA **(B)** with Tukey’s multiple comparisons test. ns = not significant, ***P* < 0.01, ****P* < 0.001, *****P* < 0.0001.

**Table S1.** **Baseline information of the patient cohorts**

The baseline information of the patient cohorts used in this article, including the OS/PFS status, gender, age, stage and grade.

| **Variables** | **TCGA** | **E-MTAB-1980** | **Javelin 101** |
| --- | --- | --- | --- |
|  | **(n = 521)** | **(n = 101)** | **(n =442)** |
| **OS Status** |  |  |  |
| Alive | 348(66.8) | 78(77.2) |  |
| Dead | 173(33.2) | 23(22.8) |  |
| **PFS Status** |  |  |  |
| Progression | 160(30.7) |  | 188(42.5) |
| No Progression | 361(69.3) |  | 253(57.5) |
| **Gender** |  |  |  |
| Female | 182(34.9) | 24(23.8) | 125 (28.3) |
| Male | 339(65.1) | 77(76.2) | 316 (71.7) |
| **Age** |  |  |  |
| median | 60 | 64 | 62 |
| range | 26-90 | 35-91 | 29-83 |
| **Stage** |  |  |  |
| Ⅰ | 262(50.3) | 66(65.3) |  |
| Ⅱ | 54(10.4) | 10(9.9) |  |
| Ⅲ | 122(23.4) | 13(12.9) |  |
| Ⅳ | 83(15.9) | 12(11.9) |  |
| **Grade** |  |  |  |
| 1 | 13(2.5) | 13(12.9) |  |
| 2 | 227(43.6) | 59(58.4) |  |
| 3 | 206(39.5) | 22(21.8) |  |
| 4 | 75(14.4) | 5(5.0) |  |
| NA |  | 2(1.9) |  |

**Table S2. Targeted sequences of siRNA and shRNA**

The targeted sequences of siRNA and shRNA used in this study, which were designed to achieve efficient gene knockdown.

| **Gene** | **Strand** | **Sequence (5′→3′)** |
| --- | --- | --- |
| shENPP2#1 | Target | CCAATCTTCGACTATGACTAT |
| shENPP2#2 | Target | GCTCCTAATAATGGGACCCAT |
| siTBK1 #1 | Sense | AGUGGAUGUUCAAAUGAGAGA |
| siTBK1 #1 | Antisense | UCUCAUUUGAACAUCCACUGG |
| siTBK1 #2 | Sense | GAGACAACAACAAGACAUAAA |
| siTBK1 #2 | Antisense | UAUGUCUUGUUGUUGUCUCCU |
| siLPAR1#1 | Sense | CGAUCUGAUCAGCAAACAAGA |
| siLPAR1#1 | Antisense | UUGUUUGCUGAUCAGAUCGAA |
| siLPAR1#2 | Sense | ACUUCUAUCUCAUGUUCAACA |
| siLPAR1#2 | Antisense | UUGAACAUGAGAUAGAAGUAG |
| siLPAR2#1 | Sense | CGCGAGUCUGUCCACUAUACA |
| siLPAR2#1 | Antisense | UAUAGUGGACAGACUCGCGGG |
| siLPAR2#2 | Sense | GGCUUCUUCUAUAACAACAGU |
| siLPAR2#2 | Antisense | UGUUGUUAUAGAAGAAGCCGA |

**Table S3. Reagents and antibodies**

The detailed information of reagents and antibodies used in this article, including the source and catalog number.

| **Type** | **Source** | **Cat No.** | **Name** |
| --- | --- | --- | --- |
| **Reagents** | MCE | HY-101772 | Ziritaxestat(GLPG1690) |
|  | MCE | HY-19329 | HA130 |
|  | MCE | HY-10358 | MK-2206 |
|  | MCE | HY-50909 | Perifosine |
|  | Beyotime | C0017 | LDH Cytotoxicity Assay Kit |
|  | Elabscience | E-CK-A325 | One-step TUNEL In Situ Apoptosis Kit |
|  | ribbio | C10310-2 | Cell-Light EdU Apollo643 In Vitro Kit |
|  | Cloud-Clone Corp | CEK623Ge | ELISA Kit for Lysophosphatidic Acid (LPA) |
|  | MCE | HY-K0301 | Cell Counting Kit-8 |
|  | MCE | HY-137862 | 1-Oleoyl lysophosphatidic acid |
| **WB antibodies** | Abcam | ab77104 | Anti-ENPP2/ATX[1F8] |
|  | Cell signaling technology | 13684S | PD-L1 (E1L3N®) XP® Rabbit mAb |
|  | Cell signaling technology | 5483S | Phospho-TBK1/NAK (Ser172) (D52C2) XP® Rabbit mAb |
|  | Cell signaling technology | 3504S | TBK1/NAK (D1B4) Rabbit mAb |
|  | Cell signaling technology | 11904S | IRF-3 (D6I4C) XP® Rabbit mAb |
|  | Cell signaling technology | 29047S | Phospho-IRF-3 (Ser396) (D6O1M) Rabbit mAb |
|  | proteintech | Cat No. 80519 | Phospho-IRF3 (Ser396) Recombinant antibody |
|  | Cell signaling technology | 4691S | Akt (pan) (C67E7) Rabbit mAb |
|  | Cell signaling technology | 4060S | Phospho-Akt (Ser473) (D9E) XP® Rabbit mAb |
|  | proteintech | Cat No. 66888 | mTOR Monoclonal antibody |
|  | proteintech | Cat No. 67778 | Phospho-mTOR (Ser2448) Monoclonal antibody |
|  | proteintech | Cat No. 10494 | GAPDH Polyclonal antibody |
|  | proteintech | Cat No. 10094 | Beta Tubulin Polyclonal antibody |
| **FC antibodies** | BioLegend | 640919 | APC Annexin V |
|  | BioLegend | 423107 | Zombie UV™ Fixable Viability Kit |
|  | BioLegend | 502511 | APC anti-human IFN-γ |
|  | BioLegend | 502908 | PE anti-human TNF-α |
|  | BioLegend | 308121 | Brilliant Violet 421™ anti-human Perforin |
|  | BioLegend | 372213 | PE/Cyanine7 anti-human/mouse Granzyme B Recombinant |
|  | BioLegend | 344741 | Brilliant Violet 605™ anti-human CD8 |
|  | BioLegend | 304061 | APC/Fire™ 750 anti-human CD45 |
|  | BioLegend | 300305 | FITC anti-human CD3 |
|  | BioLegend | 369310 | PE/Cyanine7 anti-human CD223 (LAG-3) Antibody |
|  | BioLegend | 345008 | Brilliant Violet 421™ anti-human CD366 (Tim-3) Antibody |
|  | BioLegend | 329922 | APC/Cyanine7 anti-human CD279 (PD-1) Antibody |

**Table S4. Databases and gene sets**

Curated gene sets derived from four authoritative databases, a total of 2,279 lipid metabolism-related genes were screened and used in subsequent analyses.

| **Database** | **Gene set** |
| --- | --- |
| **MsigDB** | HALLMARK_FATTY_ACID_METABOLISM |
|  |  |
| **Reactome** | REACTOME_FATTY_ACID_METABOLISM |
|  |  |
| **Kyoto Encyclopedia of Genes and Genomes** | KEGG_FATTY_ACID_METABOLISM |
|  |  |
| **Gene Ontology** | GOBP_LIPID_METABOLIC_PROCESS |
|  | GOBP_GLYCEROLIPID_METABOLIC_PROCESS |
|  | GOBP_PHOSPHATIDYLCHOLINE_METABOLIC_PROCESS |

**Table S5. Clinical and pathological characteristics of patient cohorts used for Single-cell RNA sequencing analysis.**

**(A) GSE207493**

| **Sex** | **Age** | **Tumor site** | | **Pathological histology** | | | **WHO/ISUP** | **TNM Stage** |
| --- | --- | --- | --- | --- | --- | --- | --- | --- |
| female | 58 | left | clear cell renal cell carcinoma | | | 2 | | T1aN0M0 |
| male | 63 | left | clear cell renal cell carcinoma | | 2 | | | T1bN0M0 |
| male | 27 | right | clear cell renal cell carcinoma | | 1 | | | T1aN0M0 |
| male | 51 | right | clear cell renal cell carcinoma | | 3 | | | T2aN0M0 |
| male | 40 | left | clear cell renal cell carcinoma | | 2 | | | T1bN0M0 |
| male | 47 | right | clear cell renal cell carcinoma | | 2 | | | T1aN0M0 |
| male | 55 | right | clear cell renal cell carcinoma | | 1 | | | T2aN0M0 |
| male | 29 | right | clear cell renal cell carcinoma | | 2 | | | T1aN0M0 |
| male | 40 | left | clear cell renal cell carcinoma | | 1 | | | T1aN0M0 |
| male | 51 | left | clear cell renal cell carcinoma | | 2 | | | T1bN0M0 |
| male | 60 | left | clear cell renal cell carcinoma | | 3 | | | T1bN0M0 |
| male | 58 | left | clear cell renal cell carcinoma | | 2 | | | T1bN0M0 |
| male | 53 | left | clear cell renal cell carcinoma | | 2 | | | T1aN0M0 |
| male | 47 | right | clear cell renal cell carcinoma | | 1 | | | T1bN0M0 |
| female | 60 | right | clear cell renal cell carcinoma | | 2 | | | T1aN0M0 |
| male | 66 | right | clear cell renal cell carcinoma | | 2 | | | T1bN0M0 |
| male | 48 | right | clear cell renal cell carcinoma | | 2 | | | T1aN0M0 |
| male | 55 | right | clear cell renal cell carcinoma | | 2 | | | T1bN0M0 |
| male | 57 | right | clear cell renal cell carcinoma | | 2 | | | T1aN0M0 |

**(B) Obradovic et al**

| **sample** | **Stage** | **Grade** |
| --- | --- | --- |
| CN004 | pT3a | 3 |
| CN005 | pT3a | 3 |
| CN009 | pT1a | 2 |
| CN010 | pT1a | 2 |
| CN011 | pT1a | 2 |
| CN012 | pT1a | 2 |
| CN013 | pT1a | 2 |
| CN014 | pT1a | 2 |
| CN1 | pT1a | 2 |
| CN2 | pT1a | 2 |
| CN3 | pT1a | 2 |
| CN4 | pT1a | 2 |
| CN5 | pT3a | 2 |
| CN6 | pT3a | 2 |
| CN7 | pT3a | 2 |
| CN8 | pT3a | 2 |
| CN9 | pT3a | 1 |
| CN10 | pT3a | 1 |
| CN11 | pT3a | 1 |
| CN12 | pT3a | 1 |
| CN13 | pT1a | 1 |
| CN14 | pT1a | 1 |
| CN15 | pT1a | 1 |
| CN16 | pT1a | 1 |
| CN21b | pT3aN0M1 | 4 |
| CN22b | pT3aN0M1 | 4 |
| CN23 | pT3aN0M1 | 4 |
| CN24 | pT3aN0M1 | 4 |
| CN26 | Oncocytoma | N/A |
| CN27 | Oncocytoma | N/A |
| CN28 | Oncocytoma | N/A |
| CN29 | pT1a | 2 |
| CN30 | pT1a | 2 |
| CN31 | pT1a | 2 |
| CN32 | pT1a | 2 |
| CN33 | pT3a | 3 |
| CN34 | pT3a | 3 |
| CN35 | pT3a | 3 |
| CN36 | pT3a | 3 |
| CN37 | pT1a | 2 |
| CN38 | pT1a | 2 |
| CN39 | pT1a | 2 |
| CN40 | pT1a | 2 |
